# Interstrand crosslinking of homologous repair template DNA enhances gene editing in human cells

**DOI:** 10.1038/s41587-022-01654-y

**Published:** 2023-02-27

**Authors:** Hannah I. Ghasemi, Julien Bacal, Amanda C. Yoon, Katherine U. Tavasoli, Carmen Cruz, Jonathan T. Vu, Brooke M. Gardner, Chris D. Richardson

**Affiliations:** Department of Molecular, Cellular, and Developmental Biology, University of California, Santa Barbara, CA USA

**Keywords:** Homologous recombination, Genetic engineering

## Abstract

We describe a strategy to boost the efficiency of gene editing via homology-directed repair (HDR) by covalently modifying the template DNA with interstrand crosslinks. Crosslinked templates (xHDRTs) increase Cas9-mediated editing efficiencies by up to fivefold in K562, HEK293T, U2OS, iPS and primary T cells. Increased editing from xHDRTs is driven by events on the template molecule and requires ataxia telangiectasia and Rad3-related (ATR) kinase and components of the Fanconi anemia pathway.

## Abstract (web summary)

The efficiency of homologous recombination is increased by crosslinking template DNA.

## Main

CRISPR–Cas9 enables gene editing via DNA double-strand break (DSB) generation and subsequent activation of cellular DNA repair pathways. Depending on the repair pathway that is engaged, outcomes can include disruption of the targeted gene or replacement with a new sequence that restores or introduces functionality^1^. These latter gene replacement events require the delivery of template DNA encoding new sequences to levels that support gene replacement but do not adversely affect cell viability. In translational applications, template molecules are often delivered by viral vectors. Although effective, viral workflows are expensive, difficult to scale and potentially toxic to cells. The use of nonviral template DNA is thus an appealing alternative, but the efficiency and acute toxicity of nonviral templates can be inferior to viral delivery^2^. Improved nonviral gene editing would be a powerful approach to unraveling DNA repair mechanisms, a useful laboratory technique and a promising strategy for the treatment of a multitude of diseases^3^.

**Extended Data Fig. 1:**
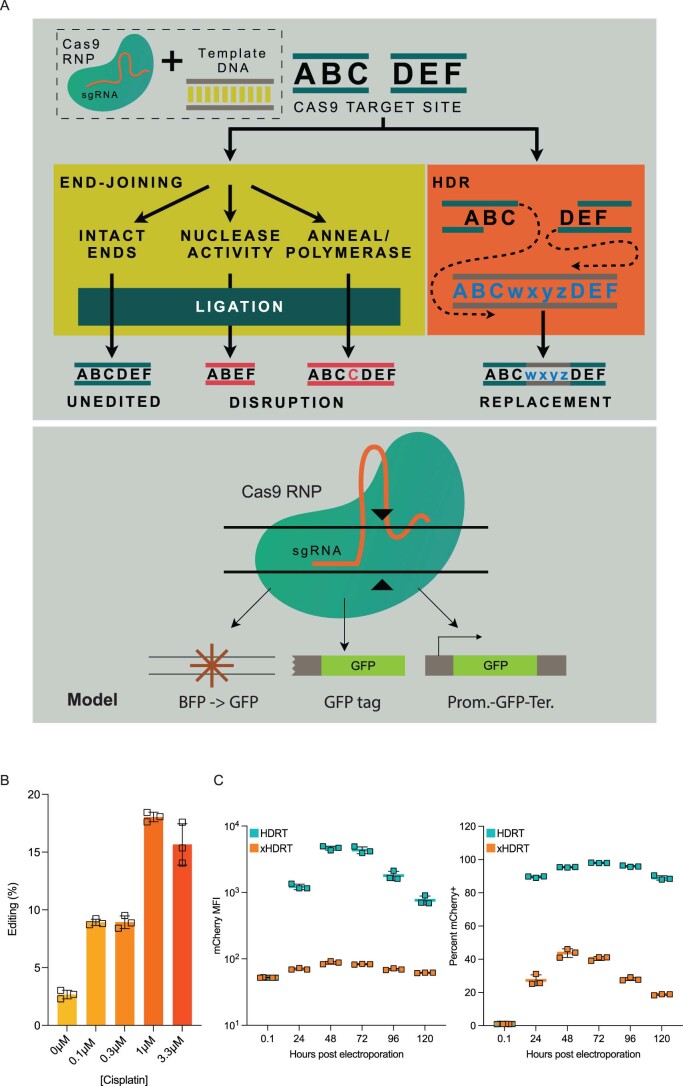
Modification of HDRTs with interstrand crosslinks increases HR during gene editing. **(a)** Top panel: Cas9 RNPs introduce a double strand DNA break (DSB) at a targeted region in the genome, which can be repaired by error prone end joining (EJ) processes that rejoin the ends of the break, or homology-directed repair (HDR) processes that resolve DSBs using sequence encoded in a separate template molecule. Bottom panel: HDR gene editing applications can be approximated using marker-based assays as diagrammed. These editing events are initiated by electroporation of Cas9, sgRNA, and HDRT into human cells, and monitored by flow cytometry or high throughput sequencing. **b)** Incorporation frequency of a pSFFV-GFP construct into the *HBB* locus of K562 cells using plasmid DNA treated with the indicated amount of cisplatin, data displayed as the mean ± SD of n = 3 biological replicates. **(c)** Transcription is inhibited from xHDRTs. Expression of dox-inducible mCherry presented both as mean fluorescence intensity (MFI) (left) and percent (right) of cells expressing mCherry encoded by uncrosslinked or xHDRT plasmid DNA. Data displayed as the mean ± SD of n = 3 biological replicates.

One high-efficiency nonviral gene editing strategy codelivers ribonucleoprotein (RNP) formulations comprising the targeted nuclease Cas9, a single guide RNA (sgRNA) and a template molecule that contains homology to the region being edited as well as the sequence to be modified or inserted^4^. These RNPs introduce DSBs at targeted regions in the genome, which are then repaired by error-prone end joining (EJ) processes that rejoin the ends of the break, or homology-directed repair (HDR) processes that resolve DSBs using sequence encoded in a separate template molecule^1^ (Extended Data Fig. 1a). The use of HDR to introduce new DNA sequence into targeted locations enables exciting gain-of-function applications^5^. Strategies to increase HDR frequency may therefore improve outcomes and decrease costs in laboratory and biomedical workflows.

Gains in nonviral HDR efficiency have been achieved through the optimization of editing reagents, including protein engineering of Cas9 and related nucleases^6^, improving the delivery of reagents into cells^7^, biophysical optimization of RNP parameters^8^, optimization of size and orientation of the homology region of template DNA^9,10^ and tethering template to editing reagents^11–13^. Parallel lines of research have focused on defining the cellular response to editing reagents with the goal of redirecting repair events through desired repair pathways^14,15^. These studies have developed key insights into DNA repair processes that underlie gene editing, but with few exceptions^16,17^, it has been hard to translate this understanding into treatments that bias DSB repair toward desirable outcomes. One limitation may be an inability to upregulate DNA repair processes that contribute to DSB repair. For example, we and others demonstrated that nonviral gene editing requires the Fanconi anemia (FA) pathway and that these FA proteins localize to DSBs^14,18,19^. However, overexpression of key FA genes failed to increase HDR beyond frequencies seen in control strains^14^.

We reasoned that adding substrates for desired DNA repair pathways to template DNA would be an effective approach to activate desired DNA repair activities. Here, we report that adding interstrand crosslinks (ICLs)—substrates for the FA DNA repair pathway—to template DNA stimulates HDR by approximately threefold on a per mole basis in human cell lines, iPS cells and stimulated T cells, without increasing mutation frequencies or altering EJ repair outcomes.

**Fig. 1:**
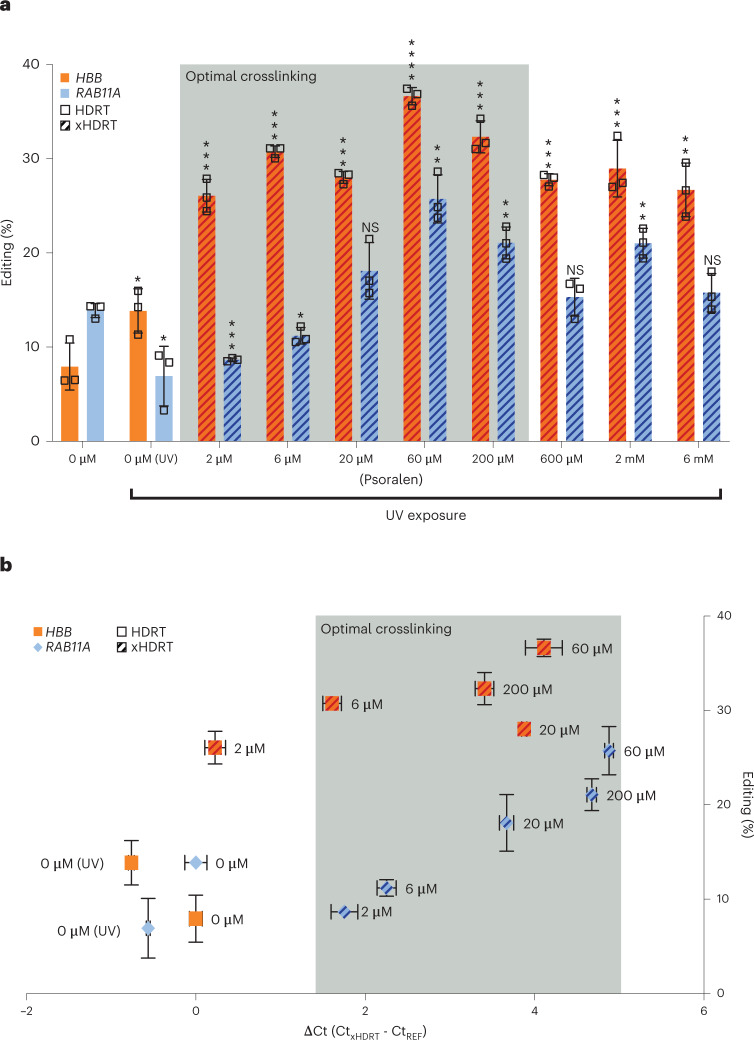
Modification of HDRTs with an optimal number of ICLs increases HDR during gene editing. **a**, Percent of cells GFP positive after editing with pSFFV-GFP (*HBB*) or N-terminal GFP fusion (*RAB11A*) constructs in human K562 myeloid leukemia cells. xHDRTs were produced by treatment with the indicated amount of psoralen and UV exposure; 0 µM, isopropanol precipitated plasmid HDRT (no UV, no psoralen). The significance of experimental conditions versus 0 µM control is displayed above columns (*P ≤ 0.05, **P ≤ 0.01, ***P ≤ 0.001, ****P ≤ 0.0001; NS, not significant). Exact *P* values reported, respectively, from left to right at the *HBB* locus are 0.04039, 0.00050, 0.00011, 0.00018, 0.00005, 0.00015, 0.00018, 0.00074 and 0.00102. Exact *P* values reported from left to right at the *RAB11A* locus are 0.02078, 0.00029, 0.01525, 0.07793, 0.00151, 0.00253, 0.31316, 0.00211 and 0.20532. **b**, Percent of cells GFP positive (*y* axis) as a function of qPCR signal loss (*x* axis), an approximation of crosslinks per unit length, for xHDRTs produced with the indicated psoralen concentration. All data displayed as the mean ± s.d. for *n* = 3 biological replicates. All data were statistically analyzed using two-tailed *t*-tests.

**Extended Data Fig. 2:**
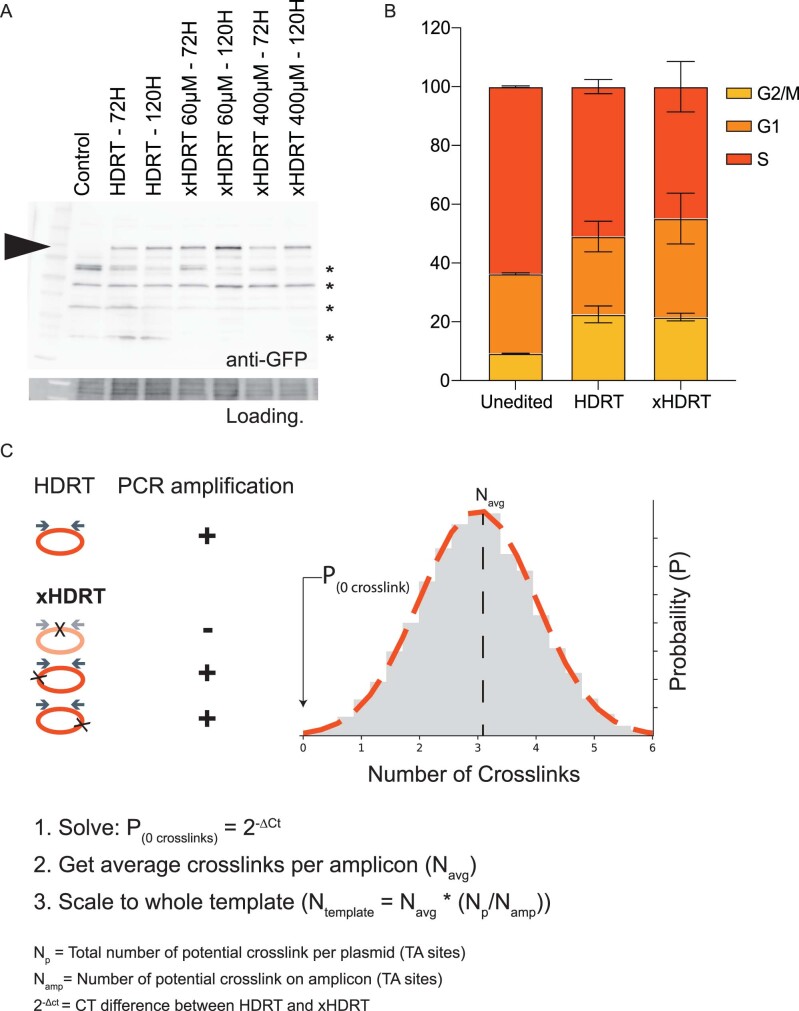
Crosslinks in xHDRTs do not alter cell cycle progression or promote off-target integration. **(a)** Western blot for GFP in K562 cells edited with xHDRTs that insert GFP at the N-terminus of *LMNB1*. Cross-reacting bands (asterisks) are shown on the blot. Predicted size of GFP-*LMNB1* indicated by solid arrow. Blot is representative of n = 3 biological replicates. **(b)** xHDRTs do not alter the cell cycle more than uncrosslinked templates. Percent of asynchronous cells edited with uncrosslinked donors or xHDRTs at the indicated point in the cell cycle. Data displayed as the mean ± SD of n = 3 biological replicates. Data were obtained by flow cytometry. **(c)** Schematic of crosslinking quantification by qPCR. Untreated (HDRT) or xHDRT molecules were amplified using PCR primers that produce a 94 bp amplicon. Cycle thresholds (Cts) were calculated for each sample and subtracted from an uncrosslinked control to obtain ∆Ct. ∆Ct numbers were used to calculate a probability of no crosslinks (P_0 crosslinks_). P_0 crosslinks_ was used to calculate an average number of crosslinks per amplicon (N_avg_). N_avg_ was scaled to the size of the xHDRT. Source data

We adapted a nonviral gene editing workflow to measure the effect of covalent modification of double-stranded HDR templates (HDRTs) on gene editing efficiency. ICLs added to an HDRT—which we refer to as xHDRTs—dramatically improve editing rates in nonviral gene editing workflows in a dose-dependent manner (Fig. 1a). ICLs are perturbing DNA lesions, which covalently tether both DNA strands together, and are repaired in human cells by replication- and transcription-coupled mechanisms^20–22^. Common crosslinking agents include psoralen, which crosslinks opposing thymines at TA sequences ^23^, and cisplatin, which crosslinks opposing guanines at GC sequences in dsDNA^24^. Both psoralen and cisplatin crosslinking reagents stimulate HDR when used to make xHDRTs, suggesting that the HDR stimulation is general to ICLs and not to a specific chemistry (Fig. 1a and Extended Data Fig. 1b). Psoralen crosslinking requires long-wave UV irradiation; thus, unreacted psoralen cannot cause genomic ICLs in cells (where no UV exposure occurs), so we prioritized the development of psoralen-derived xHDRTs. Incubation of HDRTs with varying concentrations of psoralen and 365 nm UV radiation creates xHDRTs that increase integration of GFP into the *HBB* locus of human cells approximately threefold (Fig. 1a). This effect is not caused by transcription from the template molecule, as psoralen ICLs inhibit transcription from reporter genes expressed on the xHDRT (Extended Data Fig. 1c). Nor is this effect caused by nonspecific integration of donor sequence into the genome, as xHDRTs that attach GFP to the N-terminus of *LMNB1* produce signal consistent with the fusion protein, and side products indicative of frequent off-target insertion do not appear in the edited samples (Extended Data Fig. 2a). Addition of xHDRTs to cells causes a slight enrichment of cells in the G2 phase of the cell cycle over asynchronous controls, but this is indistinguishable from cells treated with uncrosslinked templates (Extended Data Fig. 2b). We note that HDRTs containing primarily thymidine dimers^25^ caused by longwave UV radiation do not support elevated levels of HDR (Fig. 1a; 0 µM (UV)), and so increased editing is specific to ICLs and not nonspecifically caused by damaged donor DNA. Overall, xHDRTs can be used in existing gene editing workflows to boost HDR by approximately threefold on a per-mole basis.

Psoralen crosslink density is a function of the TA content of the DNA, the psoralen concentration and the UV dosage, and may thus vary between HDRTs. To estimate the optimal number of ICLs per xHDRT, we developed a qPCR-based assay that approximates the number of crosslinks within a given DNA molecule (Extended Data Fig. 2c). Using primers that amplify a 94 base pair region of the HDRT plasmid backbone, we determined the probability that at least one crosslink has been introduced in this region. We calculated the ratio (expressed as ∆Ct) of qPCR signal produced from xHDRTs generated with different psoralen concentrations or uncrosslinked templates. The editing activity of xHDRTs relative to uncrosslinked controls peaked at threefold, which occurs at a mean ∆Ct value of 4.5 (Fig. 1b). This translates to an average crosslink density of approximately 60 crosslinks per xHDRT (Extended Data Fig. 2c). These parameters were consistent for xHDRTs homologous to the *HBB* and *RAB11A* loci.

**Fig. 2:**
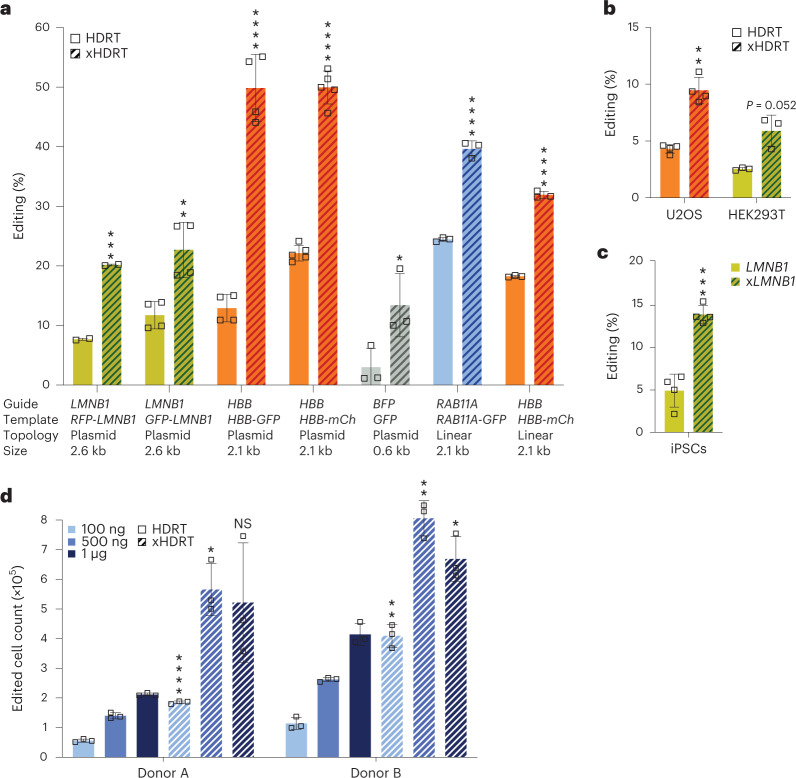
xHDRTs increase HDR in broad gene editing applications. **a**–**c**, Percent incorporation of GFP-tag (*LMNB1*, *RAB11A*), promoter–reporter (*HBB*) and SNP (*BFP*) sequences using plasmid or linear PCR-derived dsDNA of indicated sizes (homology + payload) in K562 cells (**a**). Data displayed as the mean ± s.d. of at least *n* = 2 biological replicates. Exact *P* values reported from left to right are 0.000174, 0.005566, 0.000019, 3.70 × 10^−7^, 0.043406, 0.000047 and 0.000002. Percent incorporation of a fluorophore at the *LMNB1* locus of U2OS and HEK293T cells, *P* values 0.0016, 0.05192 (**b**) or iPS cells, *P* = 0.000636 (**c**). Data displayed as the mean ± s.d. of *n* = 3 biological replicates. **d**, Absolute yield of *RAB11A*-GFP positive, viable T cells from two blood donors 168 h after editing with linear HDRT or xHDRT as gated in Extended Data Fig. 3d. Data were obtained by flow cytometry and displayed as the mean ± s.d. of *n* = 3 biological replicates; comparisons between xHDRT-edited samples versus HDRT-edited controls. Significance values are displayed above the experimental sample. *P ≤ 0.05, **P ≤ 0.01, ***P ≤ 0.001, ****P ≤ 0.0001; NS, not significant. Exact *P* values from left to right for donor A are 7.04 × 10^−6^, 0.013 and 0.117 and for donor B, 0.0013, 0.0035 and 0.01456. All data were statistically analyzed using two-tailed *t*-tests.

To define the generalizability of our xHDRTs, we tested these constructs in the context of different donor DNA topologies and sequences. xHDRTs boost gene editing in the context of linear and circular double-stranded molecules and for HDR payloads including three nucleotide SNPs (approximately fivefold), GFP-tag constructs (approximately twofold) and promoter–reporter constructs (approximately threefold) in K562 cells (Fig. 2a). To validate our approach in other human cell lines, we confirmed that xHDRTs increase HDR by approximately twofold as compared to an uncrosslinked template in additional cell lines, including U2OS and HEK293T cells (Fig. 2b). We also validated that xHDRTs stimulate HDR in iPS cells (approximately threefold; Fig. 2c), which are useful cells for regenerative medicine applications. Our overall conclusion is that xHDRTs boost gene editing in multiple payloads and target cell types.

**Extended Data Fig. 3:**
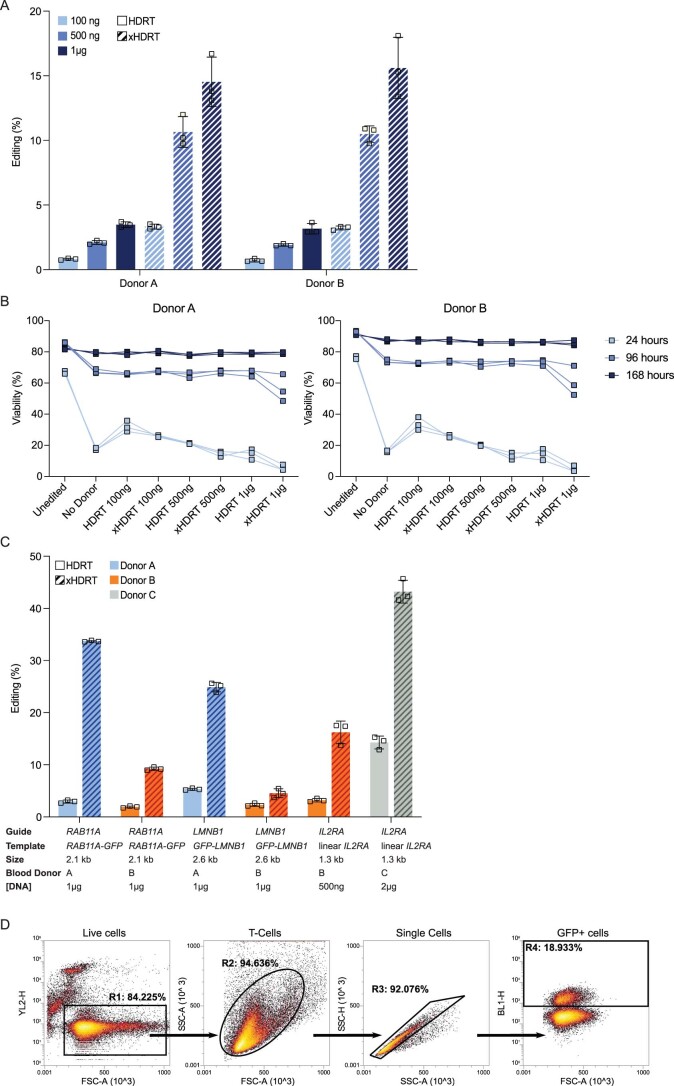
Editing, viability, and flow cytometry data supporting main Fig. 2. **(a)** Editing frequencies in two T-cell blood donors achieved using titrated concentrations of PCR-derived linear *RAB11A* template. Data shown as the mean ± SD of n = 3 biological replicates**. (b)** T-cell viability shown in two blood donors in response to titrated amounts of uncrosslinked and crosslinked PCR-derived *RAB11A*-GFP template. **(c)** Crosslinked templates support higher editing efficiencies than uncrosslinked templates in primary T cells from different blood donors and at different loci. Percent incorporation of a fluorophore at the *LMNB1, IL2RA*, or *RAB11A* loci in primary T cells. Data were obtained by flow cytometry and displayed as the mean ± SD of n = 3 biological replicates. **(d)** T-cell gating strategy. T cells were stained with PI, gated for viable cells (R1), morphology by FSC and SSC (R2), single cells (R3), and GFP + or ‘edited’ cells (R4).

We subsequently tested xHDRTs in near-therapeutic T-cell editing workflows. xHDRTs increased the final edited cell yield (gating strategy shown in Extended Data Fig. 3d) by approximately threefold compared to uncrosslinked templates (Fig. 2d). Edited cell yield measures the number of edited cells 7 days after nucleofection and thus incorporates editing percentage as well as toxicity or transient cell cycle arrest caused by editing reagents. To optimize cell yield, we tested multiple doses of crosslinked or uncrosslinked linear template. Cell yield was greatest using 500 ng of xHDRT per reaction, which yielded approximately 3.8-fold more edited T cells than the same dose of uncrosslinked template. Higher doses of xHDRT further boosted editing percentages (Extended Data Fig. 3a), but viability deficits limited cell yield (Extended Data Fig. 3b). We observed stimulation of T-cell editing by crosslinked templates at multiple loci, with multiple payload sizes, and at sites edited with frequencies ranging from 10% to over 40% (Extended Data Fig. 3c). Overall, crosslinked templates are an effective strategy to boost edited cell yield in T-cell editing workflows. Our results further indicate that cell yield is limited by toxicity caused by electroporation and donor nucleic acid; thus approaches that limit this toxicity may boost cell yield further.

**Extended Data Fig. 4:**
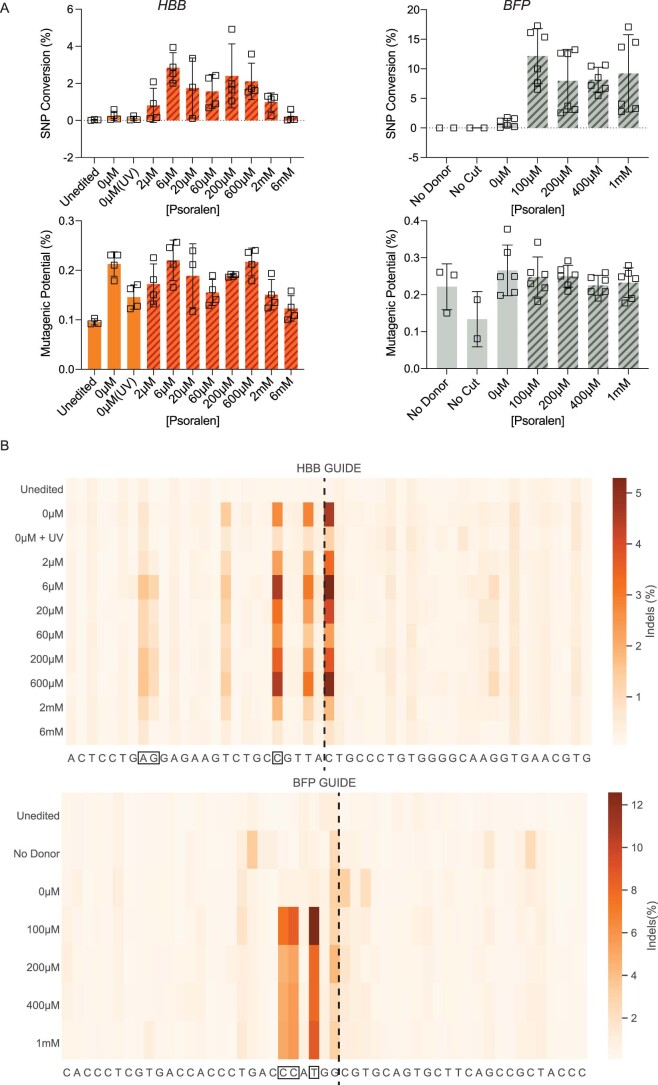
xHDRTs do not increase mutation frequency at edited loci. **(a)** xHDRTs boost SNP conversion without increasing total number of mutations in a window surrounding the cut site. SNP conversion as a function of crosslink frequency at the *HBB* E7V (left) or *BFP* (right) loci in K562 cells (top panels). Cumulative probability of a non-HDR mutation (mutagenic potential) arising within a 50 bp window surrounding the Cas9 cut site for samples edited with the indicated homology donors at *HBB* (left) or *BFP* (right) loci in K562 cells (bottom panels). Data shown as the mean ± SD from n = 4 biological replicates. **(b)** xHDRTs do not increase the mutation frequency at non-SNP bases. Heatmap showing mutation frequency at each base within a window surrounding Cas9 cut site (black dashed line) for samples edited with the indicated homology donors at *HBB* (top) or *BFP* (bottom). Nucleotides altered by successful HDR are outlined with black squares.

xHDRTs contain DNA lesions that are potentially mutagenic; however, we see no evidence that HDR using xHDRTs is more mutagenic than HDR using uncrosslinked templates. This is apparent during fluorescent tagging of endogenous genes, where we observe an approximately threefold increase in GFP cells rather than any decrease caused by frame- or codon-disrupting variants in the GFP donor sequence (Fig. 2). We further investigated mutation frequencies during SNP editing experiments and observed no increase in cumulative mutation frequencies in a window surrounding the Cas9 cut site relative to those observed during editing with RNP alone or with RNP and uncrosslinked template (Extended Data Fig. 4a). However, we note that the background mutation frequency (the noise) of our amplicon sequencing data is approximately 2 × 10^−3^ per nucleotide (Extended Data Fig. 4a, unedited). To boost the sensitivity of our assay, we focused on TA sites, which are the substrates for psoralen crosslinks and are present in the 50 bp window surrounding the *HBB* (1) and *BFP* (2) cut sites. We observe no increase in mutation frequency at these sites in xHDRTs relative to uncrosslinked controls (Extended Data Fig. 4b). Overall, we conclude that xHDRTs promote HDR without decreasing HDR fidelity.

**Fig. 3:**
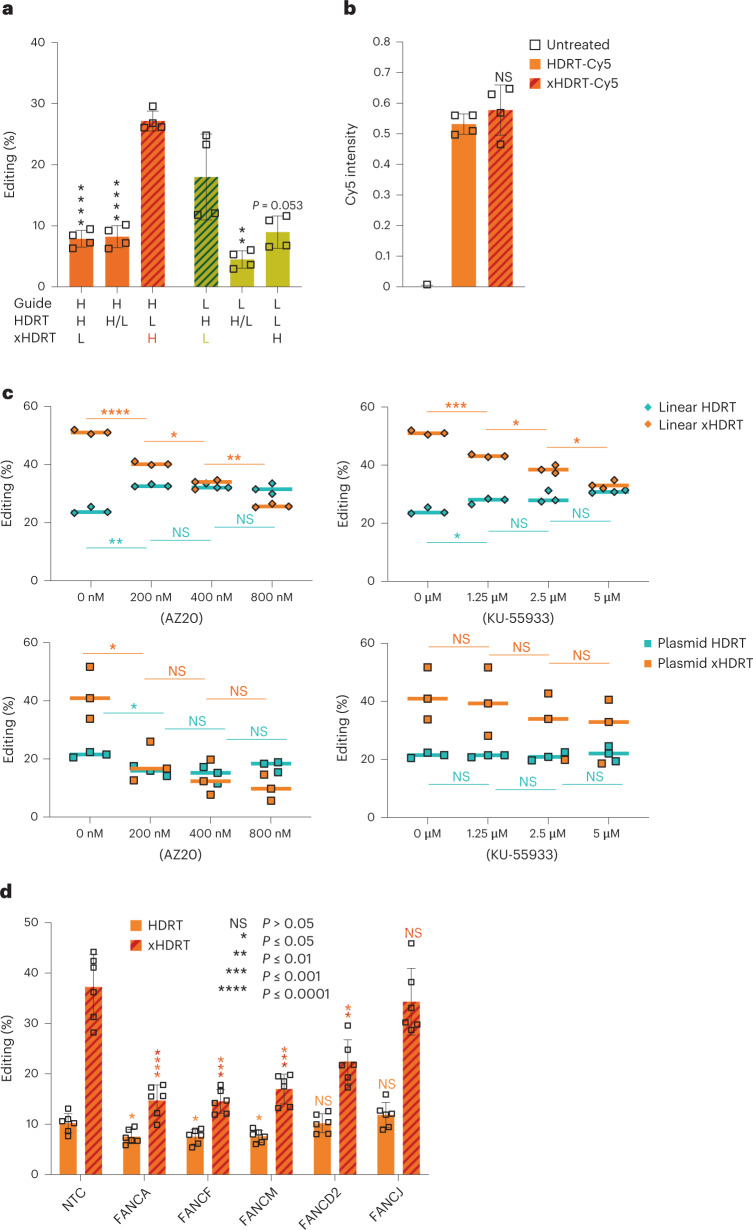
Enhanced editing from xHDRTs requires the activity of DNA repair pathways that are partially distinct from those that support HDR from uncrosslinked plasmids. **a**, ICLs stimulate HDR in *cis*. Percent incorporation of a fluorophore encoded by crosslinked (xHDRT) or uncrosslinked (HDRT) templates homologous to the *HBB* (H) and/or *LMNB1* (L) loci in K562 cells. DSBs are introduced at only one locus by providing sgRNA targeting *HBB* (H) or *LMNB1* (L). Maximal editing percentages occur when guide and xHDRT match the same locus. Data displayed as the mean ± s.d. of *n* = 4 biological replicates; comparisons between xHDRT-edited samples versus HDRT-edited controls. Exact *P* values from left to right are 1.893 × 10^−6^, 4.453 × 10^−6^, 9.305 × 10^−3^ and 5.293 × 10^−2^. **b**, ICLs do not increase the nuclear abundance of xHDRTs. Nuclear Cy5 intensity of labeled xHDRTs as compared to uncrosslinked HDRTs and untreated U2OS cells. Data displayed as the mean ± s.d. of at least *n* = 3 biological replicates; comparisons between xHDRT-treated samples versus HDRT-treated controls. **c**, xHDRT activity is ATR and ATM dependent. Percent incorporation of *HBB*-mCherry encoded by linear PCR-derived (top) or plasmid (bottom) HDRT or xHDRT in K562 cells treated with titrated concentrations of AZ20 (ATR inhibitor) or KU-55933 (ATM inhibitor). Data are shown as the median of *n* = 3 biological replicates; comparisons between edited untreated samples versus edited drug-treated controls. Exact *P* values in the top left plot from left to right in orange are 4.89 × 10^−5^, 1.10 × 10^−2^ and 8.92 × 10^−3^ and in blue, 0.003, 0.691 and 0.493. Exact *P* values (from left to right) in the top right plot in orange are 0.0003, 0.0201 and 0.0102 and in blue, 0.0161, 0.4497 and 0.2338. Exact *P* values (from left to right) in the bottom left plot in orange are 0.0250, 0.3859 and 0.5037 and in blue, 0.0143, 0.5778 and 0.2267. Exact *P* values in the bottom right plot (from left to right) in orange are 0.7903, 0.4724 and 0.8799, and in blue, 0.7229, 0.8458 and 0.6024. **d**, xHDRT activity requires components of the FA pathway. Percent incorporation of *HBB*-GFP in cells edited with HDRTs (solid) or xHDRTs (striped). Data shown as mean ± s.d. of *n* = 3 biological replicates of *m* = 2 independent knockdown cell lines; comparisons between knockdown samples versus NTC controls. Knockdown efficiency with CRISPRi is shown in Extended Data Fig. 7f. Significance values are displayed above the experimental sample, *P ≤ 0.05, **P ≤ 0.01, ***P ≤ 0.001, ****P ≤ 0.0001; NS, not significant. Exact *P* values are in order from left to right, 0.0220, 0.0001, 0.0216, 0.0001, 0.0214, 0.0002, 0.9822, 0.0013, 0.2372 and 0.4577. All data were statistically analyzed using two-tailed *t*-tests.

**Extended Data Fig. 5:**
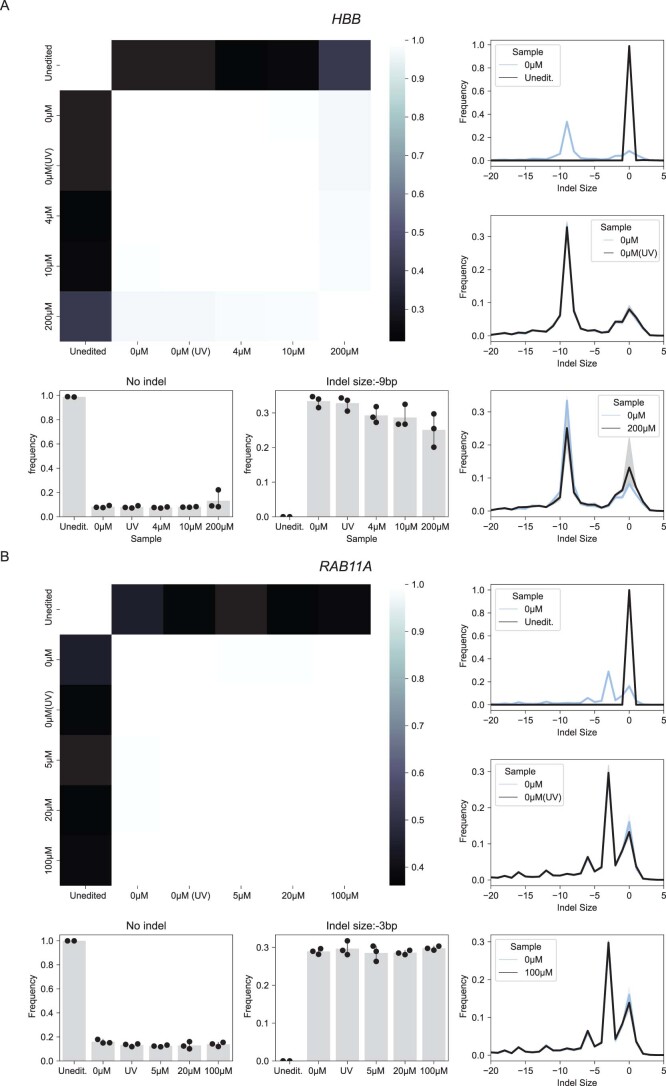
xHDRTs do not alter indel outcomes at edited loci. Clockwise from top left: (i) Pearson correlations between indel spectra for unedited or cells edited with xHDRTs produced using the indicated psoralen concentrations, (ii) frequency of indels with the indicated sizes for unedited cells vs cells edited with mock treated template, (iii) frequency of indels with the indicated sizes for cells edited with mock versus UV treated template, (iv) frequency of indels with the indicated sizes for cells edited with mock versus crosslinked template, (v) frequency of characteristic indel for cells edited using the indicated parameters, and (vi) frequency of unedited (no insertions of deletions) alleles for cells edited using the indicated parameters. All data are represented as the mean ± SD generated from n = 3 biological replicates.

xHDRTs could boost HDR through biophysical parameters, for example by altering the delivery of editing reagents or by altering the recognition of xHDRTs by cellular DNA repair pathways. To determine whether ICLs are detected in xHDRTs or trigger a cell-wide response that favors HDR, we tested if the ICL had to be present in *cis* on the homologous template molecule. We simultaneously transfected two plasmids, one containing homology to the break site and one lacking homology, with ICLs present on the homologous, nonhomologous or neither template DNA. Only ICLs on the homologous template, but not the nonhomologous template, boosted HDR at the *LMNB1* and *HB*B loci (Fig. 3a). This suggests that the xHDRT mechanism acts through local activity on the template DNA molecule and not by globally altering DNA repair pathway preferences. Consistent with this model, we observed no change in EJ outcomes at the *HBB* or *RAB11A* loci for cells edited with crosslinked or uncrosslinked templates (Extended Data Fig. 5a,b). Both loci have preferred indel outcomes of −9nt (*HBB*) or −3nt (*RAB11A*) and the relative frequency of these outcomes does not change in the presence of xHDRTs, which indicates that repair pathway preference does not change in the presence of crosslinked templates. We therefore conclude that xHDRTs specifically boost HDR frequency rather than altering global DNA repair preferences.

**Extended Data Fig. 6:**
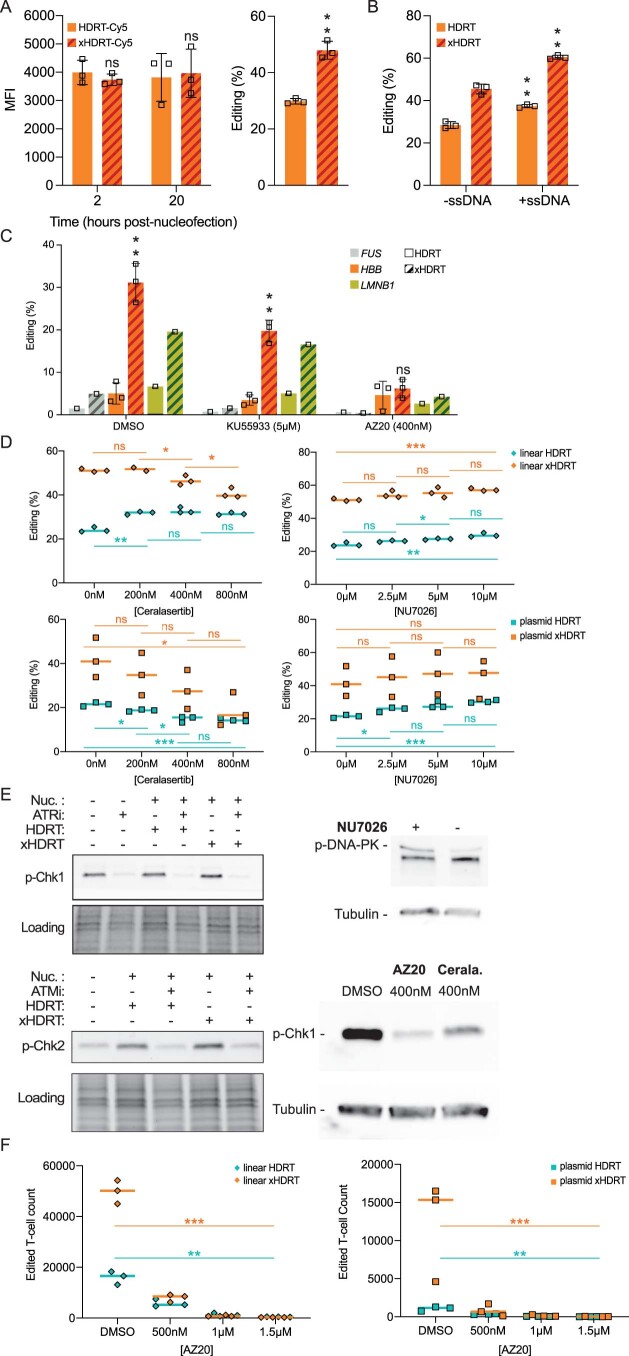
Data supporting main Fig. 3. **(a)** ICLs do not increase nuclear abundance of xHDRTs. Mean fluorescence intensity (abundance) of Cy5 labeled HDRT or xHDRT DNA in isolated nuclei shown 2- and 20-hours post-electroporation (left). Percent incorporation of an *HBB*-GFP construct (crosslinked and uncrosslinked) fused to Cy5 in K562 cells (right). Data displayed as the mean ± SD of n = 3 biological replicates; comparisons are between xHDRT-treated samples versus HDRT-treated controls. Exact p-values calculated using an unpaired two-tailed T-test at 2 and 20 hours are respectively 0.421321 and 0.841498 and the exact p-value for the editing plot is 0.006226. **(b)** xHDRTs work additively with the anionic polymer effect. Percent incorporation of a multi-kilobase (*HBB*-mCherry) construct with or without 100 pmoles of nonhomologous ssDNA. Data shown are the mean ± SD of n = 3 biological replicates; comparisons between ssDNA-treated samples versus untreated controls. Exact p-values for HDRT and xHDRT calculated using an unpaired two-tailed T-test are respectively 0.0035 and 0.0033. **(c)** xHDRT activity is ATR-dependent. Percent incorporation for GFP-tag (*FUS, LMNB1*) or promoter-reporter (*HBB*) sequences in K562 cells treated with DMSO, KU55933 (ATM inhibitor), or AZ20 (ATR inhibitor). Data displayed as the mean ± SD calculated from n = 3 biological replicates (*HBB*) or n = 1 sample (*LMNB1, FUS*); comparisons between AZ20-treated versus untreated conditions indicated by horizontal bars. Significance values are displayed above the experimental sample, * – p ≤ 0.05, ** – p ≤ 0.01, *** – p ≤ 0.001, **** – p ≤ 0.0001, ns – not significant. Exact p-values calculated using an unpaired two-tailed T-test for the *HBB* locus in DMSO, KU55933, and AZ20 are respectively 0.00274, 0.00266, and 0.53313. (**d**) xHDRT activity is ATR dependent and DNA-PK independent. Percent incorporation of *HBB*-mCherry achieved using linear PCR-derived (top) and plasmid (bottom) HDRT or xHDRT in K562 cells treated with titrated concentrations of Ceralasertib (ATR inhibitor) and NU7026 (DNA PK inhibitor). Data represented as the median calculated from n = 3 biological replicates; comparisons between treatments indicated by horizontal bars. Exact p-values calculated using an unpaired two-tailed T-test between treatments for the top left plot in orange from left to right are 0.63111, 0.04080, 0.03184 and in blue, 0.00128, 0.31019, 0.19713. Exact p-values between treatments for the top right plot in orange from left to right are 0.1055, 0.6121, 0.4400 and in blue, 0.0732, 0.0302, 0.0617. Exact p-values between the 0 µM and 10 µM conditions are 0.00078 in orange and 0.00409 in blue. Exact p-values between treatments for the bottom left plot in orange from left to right are 0.40349, 0.39957, 0.23857 and in blue 0.01990, 0.03006, 0.72860. The exact p-values between the 0 nM and 800 nM conditions are 0.02694 in orange and 0.00062 in blue. Exact p-values between treatments for the bottom right plot in orange from left to right are 0.73952, 0.85520, 0.81737 and in blue 0.01876, 0.14157, and 0.19703. The exact p-values between the 0 µM and 10 µM conditions are 0.77035 in orange and 0.00025 in blue. **(e)** Phosphatidylinositol-3 kinase-related kinases **(**PIKK) inhibitors prevent substrate phosphorylation. Western blots for phospho-Chk1, and phospho-Chk2, and phospho-DNA-PK 24 hours after the indicated treatments. Data shown is representative of n = 3 blots. **(f)** xHDRT activity is ATR-dependent in primary T cells. Absolute yield of *RAB11A*-GFP positive, viable T cells achieved using either linear PCR-derived or plasmid HDRT/xHDRT when treated with titrated concentrations of AZ20 (ATR inhibitor). Data displayed as the mean ± SD calculated from n = 3 biological replicates; comparisons between xHDRT-edited samples versus HDRT-edited controls. Exact p-values were obtained using a paired T-test comparing the DMSO control to 1.5 µM AZ20-treated T cells and are as follows, in the left plot in orange, 0.0002, and in blue, 0.0025, in the right plot in orange, 0.0006, and in blue, 0.0013. Source data

We next tested if the xHDRT effect was caused by an increased nuclear abundance of our xHDRTs. We observed no change in the nuclear abundance of xHDRTs relative to uncrosslinked controls 24 h after nucleofection in U2OS (Fig. 3b) or K562 (Extended Data Fig. 6a) cells. This indicates that ICLs do not increase the nuclear abundance of xHDRTs relative to uncrosslinked templates. It has been reported that biophysical alterations that change the size of RNP particles can improve editing outcomes^8^. We added anionic polymers (ssDNA) to editing reactions containing xHDRTs or uncrosslinked donors and observed robust increases in HDR in all contexts (Extended Data Fig. 6b), indicating that xHDRTs act independently from the anionic polymer effect. Together, these results indicate that higher levels of editing seen with xHDRTs require recognition and processing of the template molecule.

To define these mechanisms, we recovered both linear (PCR-derived) and plasmid xHDRT-edited samples into media containing small molecule inhibitors of the apical DNA repair kinases ataxia telangiectasia mutated (ATM)^26^, ataxia telangiectasia and Rad3-related (ATR) kinase^27^ and DNA-PK^28^, which have previously been inhibited to alter the frequency and type of DSB repair outcomes^29^. We found that ATR inhibition profoundly reduces (up to fivefold) the HDR frequencies of cells edited with linear or plasmid xHDRTs while modestly altering uncrosslinked HDR frequencies (Fig. 3c and Extended Data Fig. 6c,d,f). ATM inhibition reduced xHDRT HDR frequency and modestly increased linear HDRT HDR frequency, but did not change plasmid HDRT HDR frequency (Fig. 3c and Extended Data Fig. 6c). Inhibition of DNA-PK caused slight increases in HDRT and xHDRT HDR (Extended Data Fig. 6d). ATM (5 μM, KU55933), ATR (400 nM, AZ20 or Ceralasertib) and DNA-PK (5 µM, NU7026) inhibition prevented the phosphorylation of downstream targets Chk2, Chk1 and DNA-PK, respectively, confirming that kinase inhibition was effective at these doses (Extended Data Fig. 6e). ATR inhibition also decreased xHDRT HDR in primary T cells (Extended Data Fig. 6f). These observations are most consistent with a model in which multiple DNA repair pathways can use uncrosslinked template DNA but xHDRTs are processed by ATR-dependent mechanisms.

**Extended Data Fig. 7:**
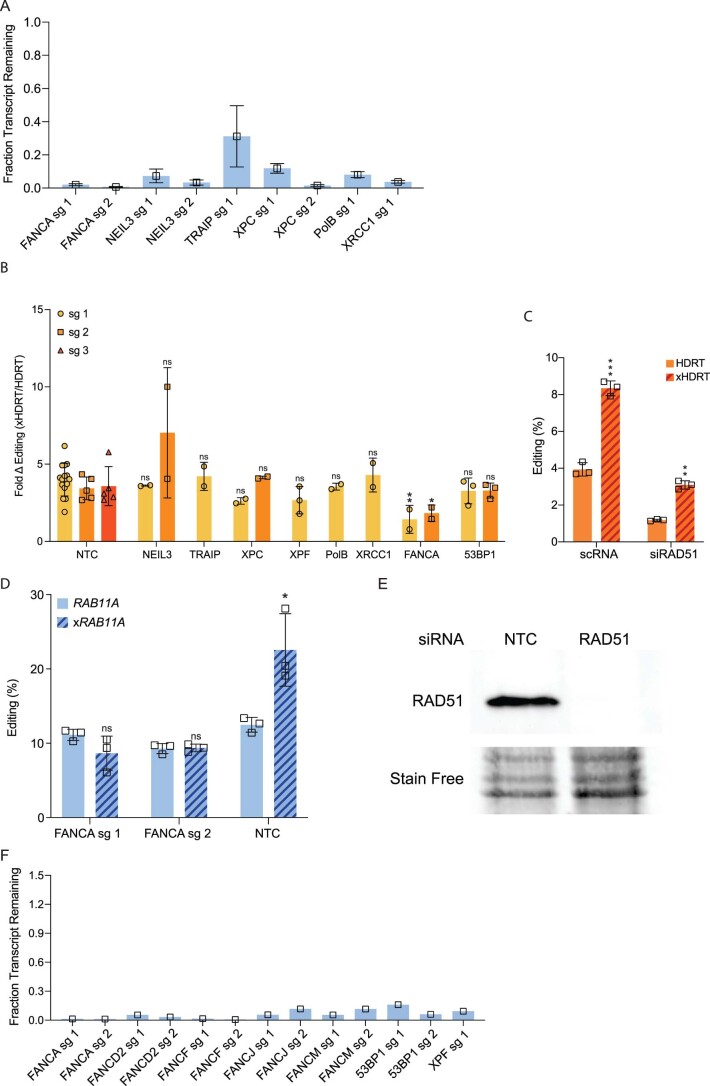
Data supporting main Fig. 3. **(a)** CRISPRi knockdown of ICL-repair genes was effective. Fraction of transcript remaining for indicated genes in CRISPRi cell lines as measured by qPCR. Data displayed as the mean ± SD of n = 3 biological replicates. **(b)** xHDRT activity is partially dependent on the Fanconi Anemia pathway. Fold change in editing supported by xHDRTs normalized against HDRT editing for the indicated knockdowns. NTC = non-targeting knockdown. Data displayed as the mean ± SD of n = 3 biological replicates. Multiple independent guides shown where indicated; comparisons between knockdown samples versus NTC controls. Exact p-values from left to right are 0.7998, 0.4740, 0.5939, 0.1243, 0.6568, 0.0813, 0.7341, 0.5234, 0.0043, 0.0141, 0.3999, 0.4156. Knockdown efficiency is shown in Extended Data Figs. 7a and 7f. **(c)** RAD51 siRNA knockdown decreases editing from both HDRT and xHDRT, data is represented as the mean ± SD calculated from at minimum n = 3 biological replicates. Exact p-values are 0.00015 for the scRNA and 0.00326 for siRAD51. **(d)** xHDRT activity is partially dependent on FANCA. Percent incorporation of a GFP-tag construct (*RAB11A*-GFP) in two independent FANCA knockdown K562 cell lines. Data displayed as the mean ± SD of n = 3 biological replicates; comparisons between knockdown samples versus NTC controls. Exact p-values were calculated using an unpaired two-tailed T-test and from left to right are 0.1511, 0.8671, 0.0247. **(e)** RAD51 siRNA-treated U2OS cells are effectively knocked down at the time of nucleofection. Western blot for RAD51 shown in U2OS cells that had been siRNA-treated for 48 hours. Data displayed as n = 1. **(f)** CRISPRi knockdown of FA pathway genes was effective. Fraction knockdown of indicated transcripts in CRISPRi cell lines as measured by qPCR. Data displayed as the mean ± SD of n = 3 biological replicates. Significance values are displayed above the experimental sample, * – p ≤ 0.05, ** – p ≤ 0.01, *** – p ≤ 0.001, **** – p ≤ 0.0001, ns – not significant. All data were statistically analyzed using unpaired, two-tailed T-tests. Source data

Due to the local effect of the ICL, we hypothesized that DNA repair factors recruited to the ICL might prime the xHDRT for use as a template. Major pathways implicated in ICL repair are the FA pathway, the nucleotide–excision repair pathway, the base-excision repair pathway and the NEIL3 glycosylase pathway^22^. We also tested the involvement of DSB-repair factors RAD51 and 53BP1 (ref. ^30^). We separately knocked down genes using stably integrated CRISPRi constructs or siRNA treatment (Fig. 3d and Extended Data Fig. 7b,c). Knockdown of FANCA substantially attenuated editing from xHDRT relative to uncrosslinked controls (Extended Data Fig. 7b,d). RAD51 inhibition reduced HDR from cells edited with uncrosslinked and crosslinked templates, indicating a role for this gene in both types of recombination (Extended Data Fig. 7c). CRISPRi and siRNA-mediated knockdowns were effective in both K562 and U2OS cells (Extended Data Fig. 7a,e,f).

To further define the involvement of the FA pathway, we individually tested knockdowns of FANCA, FANCF, FANCM, FANCJ and FANCD2. FANCA, FANCF, FANCD2 and FANCM showed a significant reduction in xHDRT-stimulated HDR, while FANCJ showed no significant reduction (Fig. 3d). These results indicate that the FA core complex and the ID2 heterodimer are important for crosslink-stimulated HDR while FANCJ helicase activities^31^ are not. We therefore conclude that the activation of the FANCD2-FANCI heterodimer contributes to increased HDR from xHDRTs.

Our previous work showed that the FA pathway is required for HDR outcomes after Cas9-mediated genome editing, but overexpression of individual FA proteins did not boost HDR frequencies^14^. Here we report that adding ICLs—a substrate of the FA pathway—to donor DNA in gene editing reactions dramatically enhances the frequency with which the template is used in HDR. This enhancement occurred in many different cell types and across a range of donors and editing reactions. We also observed that xHDRTs can be used synergistically with other strategies to boost editing efficiency, suggesting a distinct mechanism of HDR enhancement.

We also uncover the outlines of this mechanism as follows: xHDRT editing requires ATR signaling and is partially dependent on the FA pathway. The dependence on ATR, which is primarily activated through replication protein A^32^, suggests that signaling from ATR-activating nuclear structures—and not the DSB—may play a key role in specifying HDR instead of EJ repair pathways. These ATR-activating structures are unlikely to be encoded on the xHDRT, as these xHDRT molecules do not act as an agonist of ATR (Extended Data Fig. 6e; ATRi, lanes 1 and 5) and may instead comprise sites of replication stress or resected DNA. Furthermore, the requirement for ATR activity during xHDRT editing indicates that this may be a mechanistically distinct form of recombination. Therefore, the choice between EJ and HDR may include more repair options than the binary EJ/HDR model (Extended Data Fig. 1a) specifies. Overall, we favor a model in which xHDRT ICLs are uncovered and repaired during HDR itself and the repair of these lesions, and the completion of HDR, requires ATR signaling.

**Extended Data Fig. 8:**
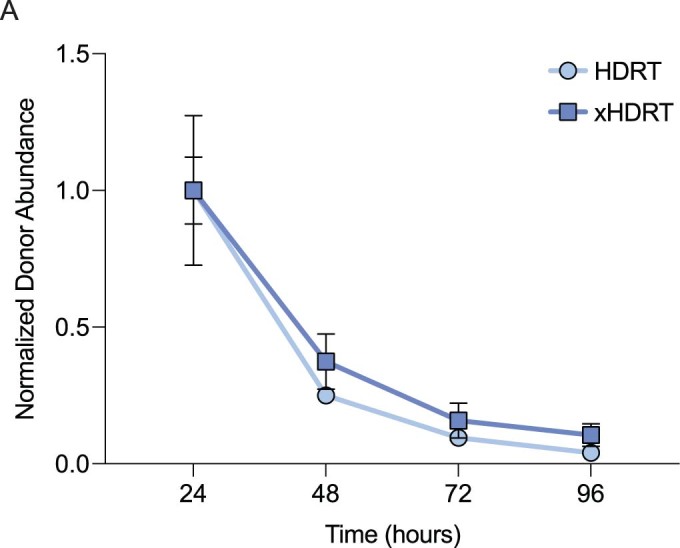
Intercellular abundance of xHDRTs and HDRTs declines at equivalent rates. **(a)** Abundance of both xHDRTs and HDRTs decreases over time in cells. qPCR plasmid quantification (AU = 2^(Ct^plasmid^-Ct^genome^)_tN_/2^(Ct^plasmid^-Ct^genome^)_t24_) at the indicated times after electroporation. Data normalized to plasmid abundance at t = 24 and is shown as the mean ± SD calculated of n = 3 biological replicates.

While our genetic results suggest the FA pathway is involved in xHDRT processing, the precise mechanism of ICL recognition remains unclear. Proposed mechanisms for FA-mediated ICL repair stipulate that DNA replication uncovers lesions, but degradation rates of HDRTs in cells are inconsistent with episomal replication of these elements (Extended Data Fig. 8a). There are additional models for transcription-coupled repair of ICLs, but components of these ICL-repair pathways (for example, XPF) are not required for xHDRT editing (Extended Data Fig. 7b). We also note that transcription itself is not required, as xHDRTs lacking any eukaryotic promoters support increased levels of HDR (Fig. 2 and Extended Data Fig. 4a;*LMNB1* and *BFP*). An intriguing possibility is therefore that xHDRT ICLs are uncovered during recombination between the DSB and the template. Validation of such a model in the context of our observation that crosslinks stimulate xHDRT recombination in *cis* would suggest that HDR is explored frequently during DSB repair and that detection of crosslinked DNA increases the likelihood that HDR will proceed. Future studies that more precisely control the location and number of crosslinks will determine if xHDRT repair occurs via known DNA repair pathways or if a new recognition mechanism is involved.

From a practical standpoint, xHDRTs support higher levels of HDR with multiple payloads and loci and in multiple cell types. We thus introduce xHDRTs as a useful tool for laboratory gene editing workflows. Using commercial reagents and the qPCR assay outlined in this manuscript to optimize crosslink density, milligram-scale xHDRT preparations can be completed in a day. Future developments of this approach may enable faster and more effective ex vivo cell therapy manufacturing.

## Methods

### Cell lines and culture

HEK293T, K562 and U2OS cells were obtained from ATCC. K562 cells were cultured in RPMI medium supplemented with 10% FBS, 1% sodium pyruvate and 100 μg ml^−1^ penicillin–streptomycin. HEK293T cells were cultured in DMEM media supplemented with 10% FBS, 1% sodium pyruvate and 100 μg ml^−1^ penicillin–streptomycin. U2OS cells were cultured in DMEM supplemented with only 10% FBS and 100 μg ml^−1^ penicillin–streptomycin. For routine passaging, adherent cells were grown to ~70% confluency, washed with 1–3 ml DPBS, and subsequently treated with 1–2 ml 0.25% trypsin-EDTA (Gibco) for 3–5 min in a 37 ºC incubator. Lifted cells were then quenched with their respective media. Cell lines were routinely tested for mycoplasma contamination using enzymatic (Lonza) and PCR-based assays (Bulldog Bio).

### qPCR quantification

Purified xHDRT or HDRT plasmids were diluted to 1 × 10^9^ and 1 × 10^8^ copies per μl based on measured concentration (Qubit BR kit, Thermo Fisher Scientific; NanoDrop or Hoechst). Diluted plasmids were analyzed by qPCR using primers annealing to the ampR gene (oCR3187, cagtgaggcacctatctcagc; oCR3188, taagccctcccgtatcgtagt). ∆Ct values were calculated between the HDRT and xHDRT molecules after the pooling of biological triplicates. ∆Cts were averaged between two concentrations of input DNA. We based our quantification on the hypothesis that at least one crosslink on the amplicon will disrupt PCR amplification. Thus, the fraction of uncrosslinked xHDRT molecules at a given psoralen concentration is equivalent to 2^ −(Ct_crosslinked_ − Ct_uncrosslinked_). We used the uncrosslinked fraction to approximate the probability mass function (code available upon request) generated by the binomial distribution for *n* = 8 AT sites and calculated the average number of crosslinks. Parameters calculated for the amplicon were scaled to obtain values for the whole template based on relative lengths.

### Cas9, RNA and HDRT preparation

*Streptococcus* *pyogenes* Cas9-NLS was obtained from the QB3 MacroLab at UC Berkeley. All sgRNAs were synthesized by Synthego as modified gRNAs with 2′-O-methyl analogs and 3′ phosphorothioate internucleotide linkages at the first three 5′ and 3′ terminal RNA residues.

All dsDNAs were derived from purified plasmid DNA from bacterial cultures containing the indicated plasmid (Qiagen Plasmid Plus) or by SPRI purification of amplified linear dsDNA.

Psoralen-mediated xHDRTs were generated by preparing dsDNA to a concentration of 100 μg ml^−1^ in 1× TE buffer in a 1.5 ml microcentrifuge tube. Psoralen (20 mM in DMSO) was then added to the reaction tube to the desired final concentration. Each reaction mixture in an open microcentrifuge tube, placed on ice, was then irradiated with long wavelength UV for 15 min in a Spectrolinker XL-1000 at 365 nm. Nonreacted psoralen was removed by isopropanol precipitation and crosslinked DNA was resuspended in 1× TE buffer.

Cisplatin-mediated xHDRTs were generated by diluting dsDNA to a concentration of 100 μg ml^−1^ in 1× TE buffer in a 1.5 ml microcentrifuge tube. Cisplatin (3.3 mM in 0.9% saline) was added to the reaction tube to the desired final concentration. The reaction was briefly vortexed and transferred to a 37 °C incubator for 1 h. Nonreacted cisplatin was removed by isopropanol precipitation and crosslinked DNA was resuspended in 1× TE.

### Cas9 RNP assembly and nucleofection

Per nucleofection, 0.50 μl of sgRNA (100 μM) were added to 1 μl of 5× RNP buffer (100 mM HEPES, 750 mM KCl, 25 mM MgCl_2_, 25% glycerol and 5 mM TCEP) in a 1.5 ml microcentrifuge tube. Cas9 protein, 1 μl (40 μM), was added to the reaction mixture and then brought up to a volume of 4 μl with nuclease-free water. dsDNA donor (1 μg), prepared at 1 μg μl^−1^, was then added to the RNP mixture. Each reaction mixture was then left to incubate for at least 5 min at room temperature to allow RNP formation. 2.5 × 10^5^ cells were collected and spun down at 500*g* for 3 min, washed once in 200 μl D-PBS and resuspended in 15 μl of nucleofection buffer (Lonza). RNP mixtures were then added to resuspended cell pellets. Reaction mixtures were electroporated in 4D Nucleocuvettes (Lonza) and later recovered to culture dish wells containing prewarmed media.

Editing was measured at defined time points after electroporation by flow cytometry (standard times are 96 and 120 h; 240 h for *RAB11A* editing—due to transcription of the plasmid). Resuspension buffer and electroporation conditions are as follows for each cell line: K562 in SF with FF-120, HEK293T in SF with DS-150, U2OS in SE with CM104, iPSC in P3 with CA-137 and T cell in P3 with EH-115.

Viability was measured at defined time points postelectroporation by flow cytometry (standard times are 24 and 48 h). Viable cells were size-gated using forward scatter (FSC) and side scatter (SSC) gating and were propidium iodide stained.

### Western blot

Approximately, 400,000 cells were lysed in 150 μl of 2× Laemmli buffer (20% glycerol, 120 mM 1 M Tris–HCl pH 6.8, 4% SDS, 0.05% bromophenol blue) containing 100 mM dithiothreitol. Samples were vortexed for 10 s at full speed, boiled for 8 min and passed three times through a 25G needle. Whole-cell extracts were separated via electrophoresis on Bio-Rad TGX gels 4–20%. Before transfer, TGX chemistry was activated for 45 s and subsequently used as a loading control. Gels were transferred onto PVDF membranes and blocked for an hour in PBS with 0.1% Tween-20 and 5% milk. Membranes were incubated overnight in primary antibodies diluted in PBS with 0.1% Tween-20 and 3% BSA. Membranes were washed in PBS with 0.1% Tween-20 three times for 10 min and incubated for an hour at room temperature with the following HRP secondary antibodies (1:5,000), Immun-Star goat anti-rabbit (GAR)-HRP conjugate (1705046) and goat anti-mouse IgG (H + L)-HRP conjugate (1706516) from Bio-Rad. Membranes were finally imaged on a Chemidoc (Image Lab, Bio-Rad). Phospho-Chk1 (1:1,000) was detected using antibody 2348 from cell signaling. Phospho-Chk2 (1:1,000) was detected using 2661 from cell signaling. GFP was detected using A11122 from Thermo Fisher Scientific (1:2,000). Phospho-DNA-PK was detected using 68716S from cell signaling (1:1,000). RAD51 was detected using 8875S from cell signaling (1:1,000).

### Dox-inducible transcription

K562 cells stably expressing the reverse tetracycline transactivator (Addgene, 26429) were nucleofected using a modified *LMNB1* donor expressing mCherry under a Tet promoter (PCR, 2070). mCherry expression from the donor plasmid was monitored by flow cytometry upon doxycycline induction (1 μg ml^−1^).

### T cell isolation and culture

T cell isolation and culture were performed as previously described^33^. Peripheral blood mononuclear cells (PBMCs) were purchased as purified PBMCs (donors A, B and C, STEMCELL). T cells of donors A, B and C were isolated from PBMCs via magnetic negative selection using an EasySep Human T Cell Isolation Kit (STEMCELL, per manufacturer’s instructions). Isolated T cells were cultured at 1 million cells per ml in ImmunoCult medium (STEMCELL) with 5% FBS (Bio-Techne), 50 μM 2-mercaptoethanol (Sigma-Aldrich) and 10 mM N-acetyl l-cysteine (Sigma-Aldrich) and were stimulated for 2 d before electroporation with anti-human CD3/CD28 magnetic dynabeads (Thermo Fisher Scientific) at a beads to cells concentration of 1:1, along with a cytokine cocktail of IL-2 at 200 U ml^−1^ (STEMCELL), IL-7 at 5 ng ml^−1^ (STEMCELL) and IL-15 at 5 ng ml^−1^ (STEMCELL). T cells were collected from their culture vessels and debeaded on a magnetic rack for several minutes. Before nucleofection, debeaded cells were centrifuged for 3 min at 500*g*, media was gently aspirated from the pellet and cells were resuspended in buffer P3 (Lonza), in which 15 μl of buffer were used per 1 million T cells.

### T cell nucleofections

RNPs were made before electroporation as described above. One million stimulated T cells were debeaded for several minutes before nucleofection and pelleted at 500*g* for 3 min. The cell pellet was then washed with DPBS. DPBS was gently aspirated from the T cell pellet and then resuspended in 15 μl of buffer P3 (Lonza). The cell suspension was then transferred to the RNP mix and thoroughly triturated. Next, the cell suspension was transferred to the well of a 20 μl nucleocuvette and immediately nucleofected using the pulse code EH115. Post nucleofection, cells were rapidly recovered in 1 ml of prewarmed media. Recovery media was composed of ImmunoCult with 5% FBS, 50 μM 2-mercaptoethanol, 10 mM N-acetyl l-cysteine and 500 U ml^−1^ IL-2. Edited T cells analyzed for viability and total cell yield were monitored daily and kept at a confluency of 1 million cells per ml.

### iPSC culture

iPSCs (AICS-0090-391) were acquired from the Allen Institute and treated essentially as described^34^. Low-passage iPSCs were thawed and cultured in 10 ml sterile-filtered mTeSR1 (STEMCELL), without antibiotic, in a 10 cm^2^ Matrigel-coated plate and grown to 70% confluency, 5 d post thaw. For routine passaging, at 70% confluency, old media was aspirated and cells were washed with 5 ml room temperature DPBS before dissociation. iPSCs were then treated with 3 ml prewarmed Accutase (Innovative Cell Technologies), and the vessel was incubated at 37 ºC for 5 min. Once cells began to detach, 3 ml DPBS was added to the Accutase-treated cells, and dissociated cells were triturated. Cells were rinsed with an additional 7 ml of DPBS for a final wash, and the dissociated cell suspension was transferred to a 15 ml conical tube and centrifuged at 500*g* for 3 min at room temperature. The supernatant was carefully aspirated, and cells were resuspended in 10 ml fresh mTeSR1 containing ROCK inhibitor (ROCKi) and counted using a Countess slide. Cells were then seeded into a Matrigel-coated six-well dish at a density of 1.5 × 10^5^ per well in 3 ml mTeSR1 containing ROCKi. Old media containing ROCKi was aspirated from each well the next day and replaced with fresh mTeSR1 without ROCKi. mTeSR1 was changed daily, and ROCKi was used for each passaging event and always removed 24 h thereafter. All cell line and primary cell work were approved by UCSB BUA2019-15.

### iPSC preassembly of Cas9 RNP

For each iPSC nucleofection, 1 μl of 5× RNP buffer (5× stock = 100 mM HEPES, 750 mM KCl, 25 mM MgCl_2_, 25% glycerol, 5 mM TCEP) and 2 μl of sgRNA (100 μM) were mixed with 1.5 μl of 40 μM Cas9 protein (QB3, MacroLab) in a microcentrifuge tube along with 1 μg of DNA and brought up to a volume of 6 μl with nuclease-free water. The RNP reaction was incubated at room temperature for 20 min.

### iPSC Cas9 RNP delivery

iPSC RNPs were made before electroporation as described above. Low-passage iPSCs, at 70% confluency, in the wells of a six-well Matrigel-coated plate were washed with 2 ml DPBS. DPBS was aspirated and then 1 ml prewarmed Accutase was added to each well. Accutase-treated cells were then incubated at 37 °C for 3–5 min. DPBS (2 ml) was added and lifted cells were triturated, followed by the addition of another 3 ml DPBS for a final wash. Lifted cells were then transferred to a 15 ml conical tube and pelleted at 500*g* for 3 min. Cells were then resuspended in 10 ml fresh mTeSR1 with ROCKi and counted using a Countess slide. Further, 4 × 10^5^ cells were aliquoted per nucleofection and pelleted at 300*g* for 5 min. Media was aspirated, and cells were washed again with DPBS. DPBS was aspirated, and cells were resuspended in 15 μl buffer P3 (Lonza). The cell suspension was then transferred to the RNP mix and thoroughly triturated in the RNP mix. The resulting cell suspension (20 μl) was carefully (avoiding the introduction of bubbles) transferred into the well of a 20 μl nucleocuvette (Lonza). Cells were immediately nucleofected using the ‘Primary Cell P3’ program and ‘CA-137’ pulse code. Post nucleofection, cells were immediately recovered into the well of a precoated 12-well Matrigel plate containing 1 ml of mTeSR1 and ROCK inhibitor. Nucleofected cells were cold-shocked for 2 d post nucleofection at 32 °C and transferred to the 37 °C incubator 3 d post nucleofection. mTeSR1 media was changed the day after nucleofection, without ROCKi. Cells were grown to 80% confluency (typically 3 d post nucleofection) and passaged using Accutase and ROCKi. Cells were then flowed at 96 and 120 h post electroporation to measure editing.

### Genomic DNA extraction (for amplicon sequencing)

Approximately, 1 × 10^6^ cells were collected 2 d post nucleofection and incubated in 200 μl of QuickExtract DNA Extraction Solution (Lucigen) at 65 °C for 15 min, 68 °C for 15 min and 95 °C for 15 min. Extracts were diluted 1:4 with dH_2_O, and insoluble cell debris was removed by centrifugation. Supernatants were then transferred to a new tube for downstream analysis.

### PCR amplification of edited regions

Edited loci were amplified using locus-specific primer pairs described in Supplementary Data using GoTaq master mix (Promega) and 200 ng of genomic DNA. The thermocycler was set for 1 cycle of 98 °C for 30 s, 35 cycles of 98 °C for 10 s, 62 °C for 10 s and 72 °C for 30 s and 1 cycle of 72 °C for 1 min. PCR amplicons (PCR1) were purified using SPRI beads, run on a 1.0% agarose gel to validate size and quantified by Qubit. Purified PCR1 DNA (100 ng) was then reamplified with PCR2 primers as listed in Supplementary Data. PCR conditions are in order as follows: 95 ºC for 2 min, 95 ºC for 30 s, 60 ºC for 20 cycles, 72 ºC for 30 s and 72 ºC for 2 min. PCR2 products were SPRI cleaned, quantified by Qubit, normalized and pooled at equimolar amounts. PCR2 pools were sequenced using 2 × 300 chemistry on a Miseq.

### Analysis of amplicon sequencing data

Reads were adapter and quality trimmed using trim_galore (version 0.6.6) and aligned to predicted amplicon sequences using bowtie2 (version 2.2.5, very sensitive local mode). Nucleotide variants at each position of the aligned reads were quantified using bcftools mpileup and bcftools call (version 1.11-1-g87d355e, m-A flags passed to bcftools call). Nucleotide variants were extracted using bcftools query in two formats as follows: all nucleotides in a 50 bp window centered on the cut site and all nucleotides in a 50 bp window centered on the cut site with HDR nucleotides removed. These values were plotted on a per nucleotide basis (Extended Data Fig. 4b) or summed to produce bar plots (Extended Data Fig. 4a).

### PCR amplification of PacBio samples

Edited or unedited samples were amplified with primers described in Supplementary Data (oCR3775–oCR3776 for *HBB*; oCR3807–oCR3808 for *RAB11A*) using GoTaq master mix (Promega) and 200 ng of gDNA. The thermocycler was set for 1 cycle of 95 °C for 2 min, 35 cycles of 95 °C for 30 s, 62 °C for 2:20 and 72 °C for 30 s and 1 cycle of 72 °C for 2 min. PCR amplicons (PCR1) were purified using SPRI beads, run on a 1.0% agarose gel to validate size and quantified by Qubit. Purified PCR1 DNA (50 ng) was then reamplified with PCR2 primers as provided in the PacBio 96 barcoded universal primers plate. PCR2 conditions were 1 cycle of 98 °C for 30 s, 20 cycles of 98 °C for 15 s, 64 °C for 15 s and 72 °C for 3 min and 1 cycle of 72 °C for 7 min. PCR2 products were SPRI cleaned, quantified by Qubit, normalized and pooled at equimolar amounts. Final preparation for sequencing was performed using the SMRTbell Express Template Prep Kit 2.0 (PacBio). Samples were sequenced on a Sequel II PacBio sequencer.

### Processing and analysis of PacBio samples

Consensus sequence calling barcode demultiplexing was performed using the parameters listed (ccs --minLength 10 --maxLength 50000 --minPasses 3 --minSnr 2.5 –minPredictedAccuracy 0.99; lima --hifi-preset SYMMETRIC-ADAPTERS --min-score 80 --min-qv 20). Resulting FASTX files were subsampled using awk to include reads that could be clearly identified as EJ by filtering out reads greater than a specific length. Length filters applied were 1,228 bp for *HBB* and 1,069 bp for *RAB11A* (amplicon length + 100 bp). Filtered FASTX files were analyzed using CRISPResso2 CRISPRessoBatch version 2.1.1. Insertion/deletion data as a function of nucleotide position (Deletion_histogram.txt) were reprocessed for display using Python (version 3+). Correlations between indel spectra for pairwise comparisons were calculated using Pearson correlations (seaborn v 0.12.0).

### Nuclear localization experiments

HDRT and xHDRT DNA were Cy5-labeled using the *Label* IT Nucleic Acid Labeling Reagents (Mirus) and used in a standard nucleofection protocol (see Cas9, RNP assembly and nucleofection, with about 1 × 10^6^ cells). At 2 and 20 h, 5 × 10^5^ cells were collected and washed in PBS. Ten percent of the cells were analyzed by flow cytometry. The rest of the samples were processed for nuclei isolation as follows: cells were resuspended in 475 μl of hypotonic buffer (20 mM Tris–HCl, pH 7.4, 10 mM NaCl and 3 mM MgCl_2_) and incubated on ice for 15 min. Ten percent NP40 (25 μl) was added, and the samples were vortexed at full speed for 20 s. Nuclei were spun for 5 min at 700*g* and resuspended in PBS. Nuclei were then assessed by flow cytometry. The quality of the nuclei was ascertained by analyzing the FSC/SSC channels (nuclei should be approximately one-third of the size of the whole cell). For microscopic analysis of nuclear localization, U2OS cells were plated on a 96-well glass bottom plate (1.5H) at a density of 1 × 10^4^ cells per well. After 20 h, cells were fixed for 10 min with 4% formaldehyde and permeabilized for 15 min with DPBS containing 0.25% Triton X100. Nuclei were then counterstained with DAPI and imaged on a spinning disk microscope. A DAPI mask was used to measure the Cy5 intensity in the nucleus.

### Small molecule inhibition

After standard nucleofection, cells (K562s or T cells) were recovered in media containing the indicated concentration of ATR inhibitor (AZ20 or Ceralasertib), ATM inhibitor (KU55933) or DNA-PK inhibitor (NU7026).

### Lentiviral packaging

Lentiviral packaging was adapted from ref. ^35^. Lentivirus was produced by transfecting HEK293T cells with standard packaging vectors using the TransIT-LT1 transfection reagent (MIR 2306, Mirus Bio). Viral supernatant was collected 48–72 h after transfection, snap-frozen and stored at −80 °C for future use.

### CRISPRi knockdown

Lentiviral constructs encoding gRNAs targeting FANCA, FANCD2, FANCF, FANCJ, FANCM, 53BP1, NEIL3, XPC, XPF, POLB, TRAIP, XRCC1 or a nontargeting sequence (Supplementary Data) were separately transduced into K562 cells containing dCas9–KRAB (clone K1e^14^). The resulting cell populations were selected for homogeneity using puromycin (1 μg ml^−1^). Pooled knockdown cell populations were tested as described in the manuscript, and knockdowns were validated by qPCR.

### qPCR for CRISPRi cell lines

For qPCR, between 2.5 × 10^5^ and 1 × 10^6^ CRISPRi cells were collected. RNA was extracted using RNeasy Mini Kits (Qiagen). RNA was quantified by nanodrop, and cDNA was produced from 1 μg of purified RNA using the iScript Reverse Transcription Supermix for room temperature–qPCR (Bio-Rad). qPCR reactions were performed using the SsoFast Universal SYBR Green Supermix (Bio-Rad) in a total volume of 10 μl with primers at final concentrations of 500 nM. The thermocycler was set for 1 cycle of 95 °C for 2 min, and 40 cycles of 95 °C for 2 s and 55 °C for 8 s. Fold enrichment of the assayed genes over the housekeeping control *ACT1B* locus was calculated using the 2^−ΔΔ*C*^*T* method essentially as described.

### siRNA experiments

Between 1 × 10^5^ and 2 × 10^5^ U2OS cells were lipofectamine transfected with 50 pmols of either RAD51 siRNA (Ambion, s531930) or an NTC siRNA (Thermo Fisher Scientific, 4390843). Cells were siRNA treated for 48 h, nucleofected, and an aliquot of cells was collected for western blot at the time of nucleofection. Cells were collected for flow cytometry 96 h post nucleofection.

### Cell cycle experiments

Cell cycle analysis was performed using Click-iT EdU Alexa Fluor 647 Flow Cytometry Assay Kit (Thermo Fisher Scientific, C10424) with the following modifications: cells were pulse-labeled with EdU at 10 μM final for 30 min, fixed in 4% formaldehyde for 10 min, washed twice with PBS containing 1% BSA and permeabilized for 15 min with PBS containing 0.5% Triton X-100. Click iT reaction was carried out following manufacturer instructions. After three washes with PBS containing 1% BSA, cells were treated for 30 min with RNase A and stained for 10 min with propidium iodide and run on the flow cytometer.

### Pairwise comparisons between data

Statistical comparisons in Figs. 1–3 and Extended Data Figs. 6 and 7 and elsewhere in the paper were made using unpaired two-tailed *t*-tests with equal variance or unpaired two-tailed *t*-tests with unequal variance, where specified by the *F*-test of equality of variances. Nucleofections in Extended Data Fig. 6f were split into different drug treatment wells, and so comparisons were made using paired two-tailed *t*-tests.

### Reporting summary

Further information on research design is available in the Nature Portfolio Reporting Summary linked to this article.

## Online content

Any methods, additional references, Nature Portfolio reporting summaries, source data, extended data, supplementary information, acknowledgements, peer review information; details of author contributions and competing interests; and statements of data and code availability are available at 10.1038/s41587-022-01654-y.

### Supplementary information

Reporting Summary

Supplementary Data Reagents used in the paper.

Supplementary Code Code used to calculate ICLs/plasmid.

### Source data

Source Data Extended Data Figs. 2, 6 and 7 Unprocessed western blots.

## Data Availability

Amplicon sequencing data have been deposited in the SRA with the BioProject accession number PRJNA913199. Other relevant data are available from the corresponding authors upon reasonable request. Source data are provided with this paper.

## Code availability

All relevant code supporting the findings of this study are available in the Supplementary Information section.
