# Supplementary material for: Interstrand crosslinking of homologous repair template DNA enhances gene editing in human cells
Source: Nat Biotechnol. 2023 Feb 27;41(10):1398–404. doi: 10.1038/s41587-022-01654-y (PMC10460463; doi:10.1038/s41587-022-01654-y)
Supplement: Supplementary file 1 — Reporting Summary [file 41587_2022_1654_MOESM1_ESM.pdf]

nature portfolio

Corresponding author(s): Chris Richardson

Last updated by author(s): October 12, 2022

Reporting Summary

Nature Portfolio wishes to improve the reproducibility of the work that we publish. This form provides structure for consistency and transparency in reporting. For further information on Nature Portfolio policies, see our Editorial Policies and the Editorial Policy Checklist.

Statistics

For all statistical analyses, confirm that the following items are present in the figure legend, table legend, main text, or Methods section.

n/a	Confirmed	
☐	☒	The exact sample size (*n*) for each experimental group/condition, given as a discrete number and unit of measurement
☐	☒	A statement on whether measurements were taken from distinct samples or whether the same sample was measured repeatedly
☐	☒	The statistical test(s) used AND whether they are one- or two-sided<br>*Only common tests should be described solely by name; describe more complex techniques in the Methods section.*
☒	☐	A description of all covariates tested
☒	☐	A description of any assumptions or corrections, such as tests of normality and adjustment for multiple comparisons
☐	☒	A full description of the statistical parameters including central tendency (e.g. means) or other basic estimates (e.g. regression coefficient) AND variation (e.g. standard deviation) or associated estimates of uncertainty (e.g. confidence intervals)
☐	☒	For null hypothesis testing, the test statistic (e.g. *F*, *t*, *r*) with confidence intervals, effect sizes, degrees of freedom and *P* value noted<br>*Give P values as exact values whenever suitable.*
☒	☐	For Bayesian analysis, information on the choice of priors and Markov chain Monte Carlo settings
☒	☐	For hierarchical and complex designs, identification of the appropriate level for tests and full reporting of outcomes
☐	☒	Estimates of effect sizes (e.g. Cohen's *d*, Pearson's *r*), indicating how they were calculated

Our web collection on statistics for biologists contains articles on many of the points above.

Software and code

Policy information about availability of computer code

Data collection	Flow cytometry - Attune cytometer (v3:1); Blots - ChemiDoc™ MP, qPCR - BioRad CFX96; Amplicon Sequencing - Illumina Miseq or PacBio; DNA concentration measurement - Qubit 3.0, HOESCHT, or Nanodrop; Microscopy - Cell Profiler.
Data analysis	FACS - FlowJo (v10.7.1); Amplicon Sequencing - trim_galore (v0.6.6), bowtie2 (v2.2.5), bcftools mpileup (v1.11-1-g87d355e), CRISRPRessoBatch 2.1.1; Graphing - GraphPad Prism (v8.4.3). Data Analysis - Python (v3.7.12).

For manuscripts utilizing custom algorithms or software that are central to the research but not yet described in published literature, software must be made available to editors and reviewers. We strongly encourage code deposition in a community repository (e.g. GitHub). See the Nature Portfolio guidelines for submitting code & software for further information.

Data

Policy information about availability of data

All manuscripts must include a data availability statement. This statement should provide the following information, where applicable:

- Accession codes, unique identifiers, or web links for publicly available datasets
- A description of any restrictions on data availability
- For clinical datasets or third party data, please ensure that the statement adheres to our policy

Amplicon sequencing reads have been uploaded to SRA as BioProject PRJNA913199.

Human research participants

Policy information about studies involving human research participants and Sex and Gender in Research.

Reporting on sex and gender	Blood Donor A : Sex - Female<br>Blood Donor B : Sex - Male<br>Blood Donor C : Sex - Male
Population characteristics	Blood Donor A : Age - 29 years old; Blood Type - A-; Ethnicity - Caucasian; Smoker<br>Blood Donor B : Age - 30 years old; Blood Type - O+; Ethnicity - Caucasian; Non-smoker<br>Blood Donor C : Age - 45 years old; Blood Type - B+; Ethnicity - Caucasian; Smoker<br>All blood donors passed viral testing (HIV 1/2, HEP B/C)
Recruitment	*Describe how participants were recruited. Outline any potential self-selection bias or other biases that may be present and how these are likely to impact results.*
Ethics oversight	Product was collected using consent forms and protocols approved by either an Institutional Review Board, the Food and Drug Administration, the U.S. Department of Health and Human Services, and/or an equivalent regulatory authority.

Note that full information on the approval of the study protocol must also be provided in the manuscript.

Field-specific reporting

Please select the one below that is the best fit for your research. If you are not sure, read the appropriate sections before making your selection.

☒ Life sciences ☐ Behavioural & social sciences ☐ Ecological, evolutionary & environmental sciences

For a reference copy of the document with all sections, see nature.com/documents/nr-reporting-summary-flat.pdf

Life sciences study design

All studies must disclose on these points even when the disclosure is negative.

Sample size	No calculations were performed to determine sample size. A minimum of two biological replicates was adhered to throughout the paper..
Data exclusions	FANCL data was excluded from the manuscript. Technical failure (microbial contamination, failed CRISPRi knockdown) were predefined criteria for rejection.
Replication	Cells were separated into multiple stocks, nucleofected, recovered and analyzed separately (usually in biological triplicate). All data replicated and are shown in the manuscript.
Randomization	Randomization is not applicable.
Blinding	Investigators were not blinded to experimental outcomes.

Reporting for specific materials, systems and methods

We require information from authors about some types of materials, experimental systems and methods used in many studies. Here, indicate whether each material, system or method listed is relevant to your study. If you are not sure if a list item applies to your research, read the appropriate section before selecting a response.

Materials & experimental systems

n/a	Involved in the study	
☐	☒	Antibodies
☐	☒	Eukaryotic cell lines
☒	☐	Palaeontology and archaeology
☒	☐	Animals and other organisms
☒	☐	Clinical data
☒	☐	Dual use research of concern

Methods

n/a	Involved in the study	
☒	☐	ChIP-seq
☐	☒	Flow cytometry
☒	☐	MRI-based neuroimaging

Antibodies

Antibodies used	Phospho-Chk1 (1:1000) was detected using antibody #2348 from Cell Signaling. Phospho-Chk2 (1:1000) was detected using #2661 from Cell Signaling. GFP was detected using #A11122 from ThermoFisher (1:2000). Phospho-DNA-PK was detected using #68716S from Cell Signaling (1:1000). RAD51 was detected using #8875S from Cell Signaling (1:1000). Secondary antibodies used were Immun-Star Goat Anti-Rabbit (GAR)-HRP Conjugate #1705046 from BIO-RAD (1:5000) and Goat Anti-Mouse IgG (H+L)-HRP Conjugate #1706516 from BIO-RAD (1:5000).
Validation	Antibodies were newly purchased and used per manufacturer's discretion. No additional validation was performed.

Eukaryotic cell lines

Policy information about cell lines and Sex and Gender in Research

Cell line source(s)	HEK293T, U2OS, and K562 cells - ATCC; UMSCC1 cells - Fanconi Anemia Research materials repository; PBMCs - STEMCELL, iPS cells - Allen Institute.
Authentication	Cell lines were authenticated by distributors using STR profiling.
Mycoplasma contamination	All cell lines tested negative for mycoplasma and are routinely re-tested.
Commonly misidentified lines (See ICLAC register)	None of the cell lines used in this study are listed in the ICLAC database.

Flow Cytometry

Plots

Confirm that:

☒ The axis labels state the marker and fluorochrome used (e.g. CD4-FITC).

☒ The axis scales are clearly visible. Include numbers along axes only for bottom left plot of group (a 'group' is an analysis of identical markers).

☒ All plots are contour plots with outliers or pseudocolor plots.

☒ A numerical value for number of cells or percentage (with statistics) is provided.

Methodology

Sample preparation	HEK293T and U2OS cells (ATCC) and UMSCC1 cells (Fanconi Anemia Research material repository) were washed with 1 mL DPBS in their final culturing vessels (six-well plate), trypsinized with 0.25 mL 0.05% trypsin-EDTA (Gibco) for 3-5 minutes in a 37ºC incubator, and quenched with 1mL of DMEM supplemented with 10% FBS. iPS cells (in either a six-well or a 12-well plate) were respectively treated with 0.25 or 0.50 ml Accutase (Innovative Cell Technologies), incubated for 3-5 minutes at 37ºC, and later triturated with 1 mL DBPS and transferred into a 15 mL conical tube. An additional mL of DPBS was added to the plate for a final wash and transferred into a 15 mL conical. iPS cells were pelleted at 500g for 3 minutes, and then resuspended in 1 mL mTeSR1. T-cells were diluted 1:5 with DPBS + 1% FBS containing propidium iodide (PI). All remaining cell lines were minimally processed (resuspended in standard media). All cells were analyzed by flow cytometry on an Attune NxT.
Instrument	Attune NxT
Software	FlowJov10.7.1
Cell population abundance	Analytical flow had >10,000 cells.
Gating strategy	Viable PI-stained T-cell populations were distinguished using an FSC-A/YL2-H gate. All live cell populations were distinguished using an FSC-A/SSC-A gate, then single cells were distinguished using an SSC-A/SSC-H gate, gated off of the live cell gate. Downstream analyses were performed on the viable single cell population (cell lines).

☒ Tick this box to confirm that a figure exemplifying the gating strategy is provided in the Supplementary Information.
