# Supplementary figures and images for: Interstrand crosslinking of homologous repair template DNA enhances gene editing in human cells
Source: Nat Biotechnol. 2023 Feb 27;41(10):1398–404. doi: 10.1038/s41587-022-01654-y (PMC10460463; doi:10.1038/s41587-022-01654-y)

Fig S2-A

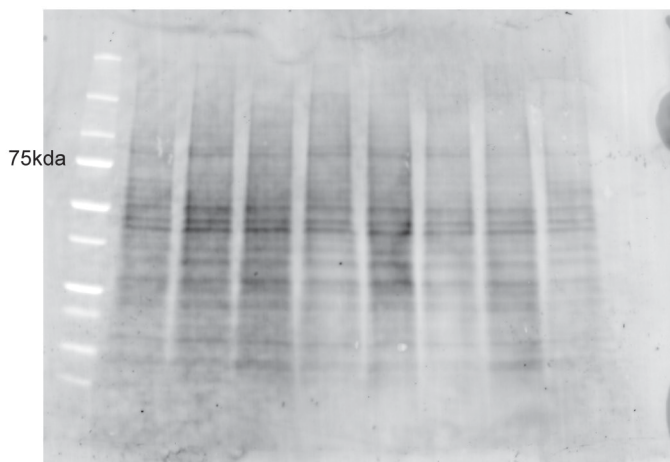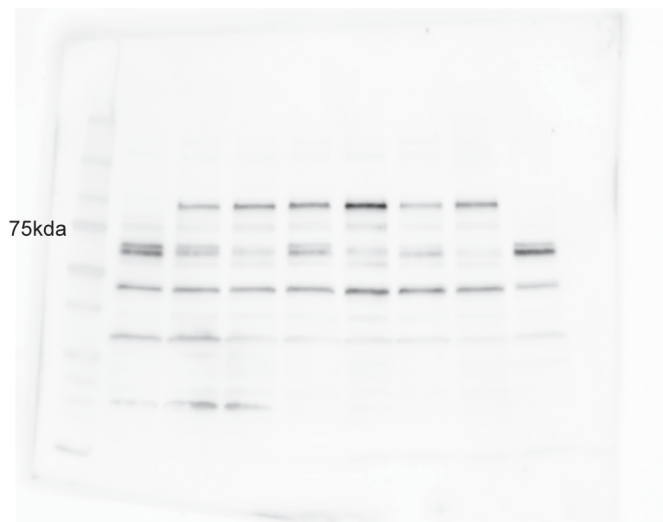

Fig S6-E-LEFT

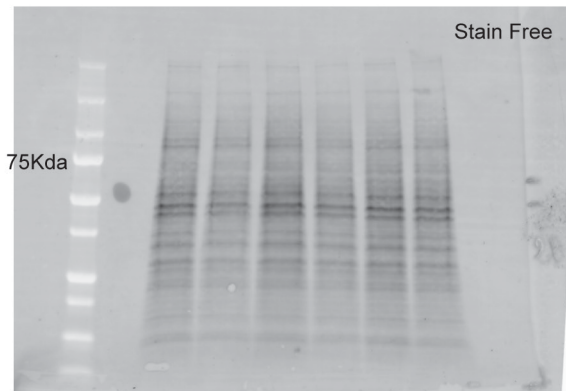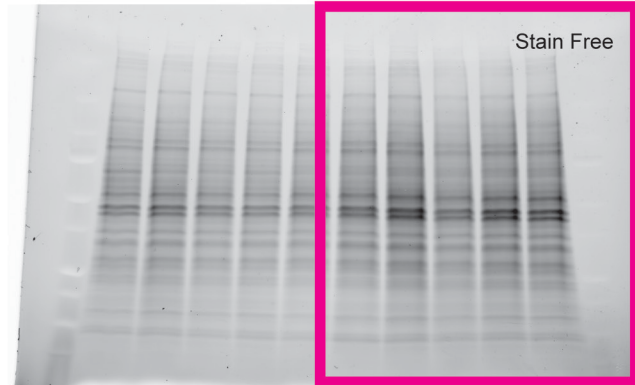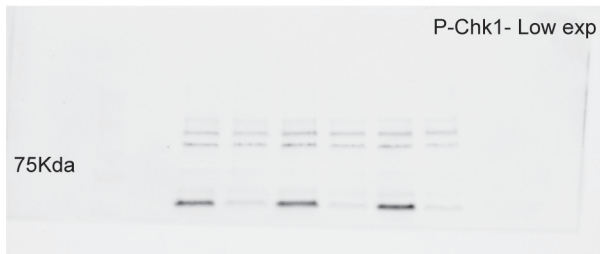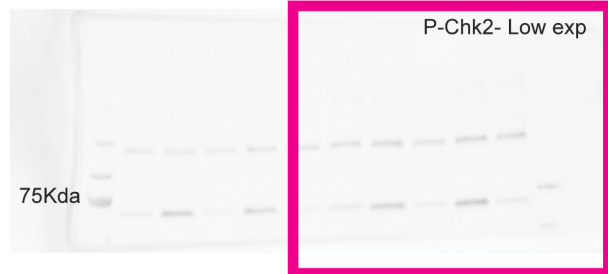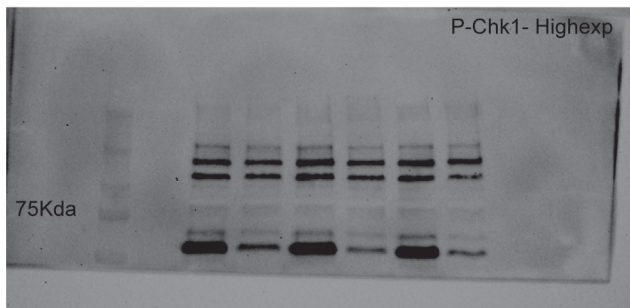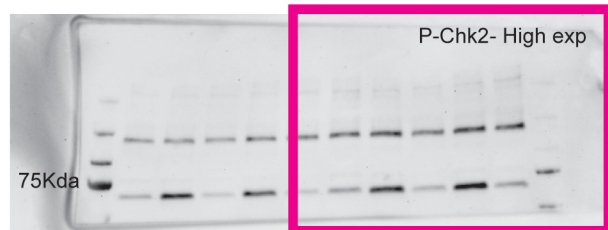

Fig S6-E-RIGHT

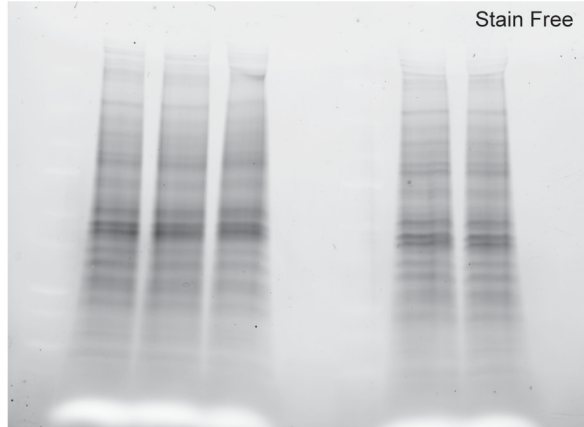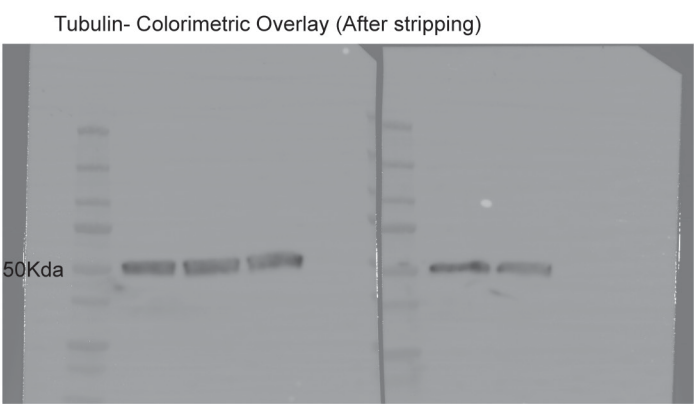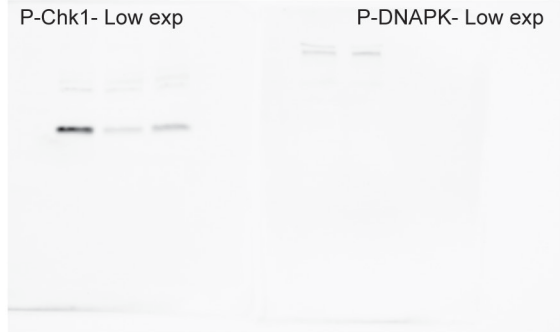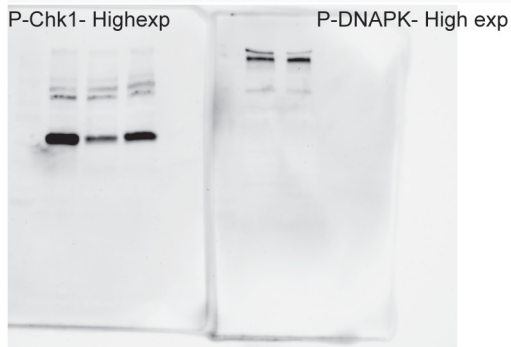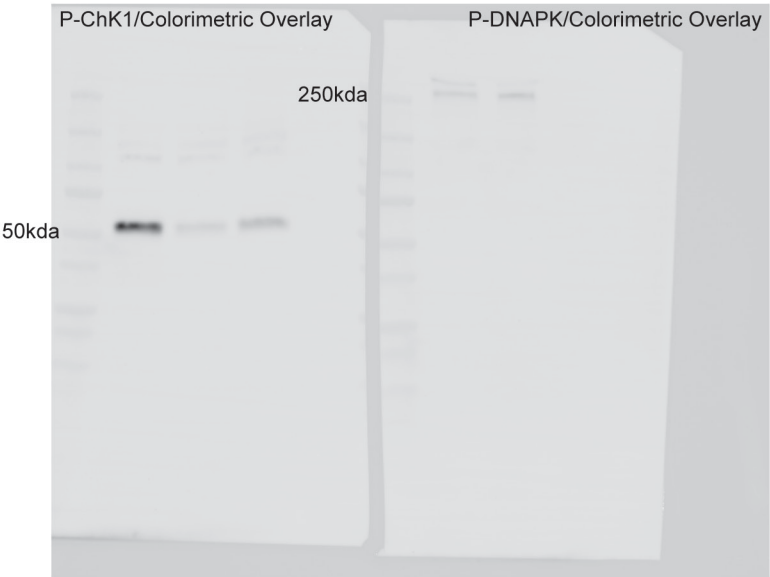

Fig S7

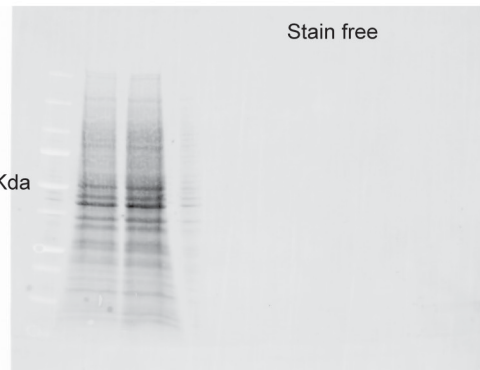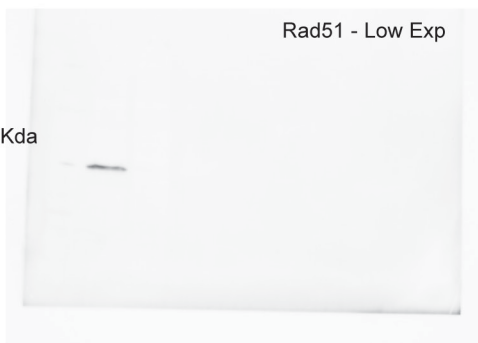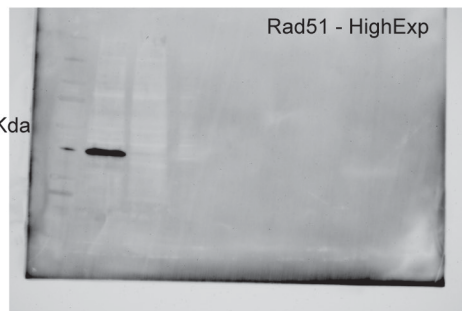

Supplement: Supplementary file 4 — Unprocessed western blots. [file 41587_2022_1654_MOESM4_ESM.pdf]
